# Promising Antioxidative Effect of Berberine in Cardiovascular Diseases

**DOI:** 10.3389/fphar.2022.865353

**Published:** 2022-03-07

**Authors:** Na An, Guoxia Zhang, Yingjian Li, Chao Yuan, Fan Yang, Lijing Zhang, Yonghong Gao, Yanwei Xing

**Affiliations:** ^1^ Guang’anmen Hospital, Chinese Academy of Chinese Medical Sciences, Beijing, China; ^2^ Key Laboratory of Chinese Internal Medicine of Ministry of Education, Dongzhimen Hospital, Beijing University of Chinese Medicine, Beijing, China; ^3^ Beijing Anzhen Hospital, Capital Medical University, Beijing, China; ^4^ Dezhou Second People’s Hospital, Dezhou, China; ^5^ Department of Cardiology, Dongzhimen Hospital, Beijing University of Chinese Medicine, Beijing, China

**Keywords:** Berberine, cardiovascular disease, reactive oxygen species, coronary atherosclerosis, myocardial infarction/reperfusion

## Abstract

Berberine (BBR), an important quaternary benzylisoquinoline alkaloid, has been used in Chinese traditional medicine for over 3,000 years. BBR has been shown in both traditional and modern medicine to have a wide range of pharmacological actions, including hypoglycemic, hypolipidemic, anti-obesity, hepatoprotective, anti-inflammatory, and antioxidant activities. The unregulated reaction chain induced by oxidative stress as a crucial mechanism result in myocardial damage, which is involved in the pathogenesis and progression of many cardiovascular diseases (CVDs). Numerous researches have established that BBR protects myocardium and may be beneficial in the treatment of CVDs. Given that the pivotal role of oxidative stress in CVDs, the pharmacological effects of BBR in the treatment and/or management of CVDs have strongly attracted the attention of scholars. Therefore, this review sums up the prevention and treatment mechanisms of BBR in CVDs from *in vitro*, *in vivo*, and finally to the clinical field trials timely. We summarized the antioxidant stress of BBR in the management of coronary atherosclerosis and myocardial ischemia/reperfusion; it also analyzes the pathogenesis of oxidative stress in arrhythmia and heart failure and the therapeutic effects of BBR. In short, BBR is a hopeful drug candidate for the treatment of CVDs, which can intervene in the process of CVDs from multiple angles and different aspects. Therefore, if we want to apply it to the clinic on a large scale, more comprehensive, intensive, and detailed researches are needed to be carried out to clarify the molecular mechanism and targets of BBR.

## 1 Introduction

Cardiovascular disease (CVD) is the leading cause of mortality in the world. The World Heart Federation reported that the number of people died of CVD is up to 17.3 million every year. It is estimated that by 2030, the number of deaths due to CVD will increase to 23.6 million (Smith et al., 2012; Hao et al., 2017). CVD accounts for up to 40% of the disease mortality in China, making it the first cause of death and premature death among Chinese residents. China and India have the highest burden of CVD in the world (Zhou et al., 2016; Zhao et al., 2019). Because of the unsatisfied demands of Western medicine for the management of CVD, some clinicians have focused on traditional Chinese medicine (TCM) to find out what role it can play in the prevention and treatment of CVD. A large number of studies in this area, both basic and clinical, have attracted increasing attention from the cardiovascular community (Hao et al., 2017). For many centuries, BBR has been widely used to treat various intestinal infections and fungal infections due to its well-known and powerful antimicrobial effects. In addition, BBR can also act on the cardiovascular system, such as regulating blood lipids and sugar, anti-arrhythmia, vasodilation effects (Shaffer, 1985; Marin-Neto et al., 1988). BBR may be cardioprotective by controlling oxidative stress and reducing injury to the myocardium, which protects the heart, according to recent *in vitro* and *in vivo* researches (Table 1). To promote pharmacological research, development, and utilization of BBR, this review describes the preventive effects of BBR in CVDs from the perspective of ROS.

**TABLE 1:** Detected studies reporting potential antioxidative stress effects of berberine in CVDs.

Application	Experiment	Intervention measures	Model	Target	References
Coronary atherosclerosis	*In vivo*	Berberine	C57BL/6 mice and ApoE^−/−^ mice	Inflammatory and oxidative markers (NF-κB, ICAM-1, IL-6, i-NOS)	Feng et al. (2017)
	*In vivo*	Berberine	Apoe^−/−^ mice with hyperhomocysteinemia	Peroxisome proliferator-activated receptor-γ (PPARγ)	Li H. et al. (2016)
	*In vivo*	Berberine	Male SHR and WKY rats	AMPK, endoplasmic reticulum (ER) stress, COX-2	Liu et al. (2015)
	*In vivo*	Berberine	Male C57BLKS/J-Leprdb/Leprdb mice	AMPK	Jeong et al. (2009)
	*In vitro*	Berberine	The murine cell line J774A.1	AMPK/mTOR	Fan et al. (2015)
	*In vitro*	Berberine	Human umbilical vein endothelial cells	LDL, oxLDL	Hsieh et al. (2007)
	*In vitro*	Berberine	Human peripheral blood mononuclear cells	NLRP3 inflammasome and IL-1β	Jiang et al. (2017)
	*In vitro*	Berberine	The human acute monocytic leukemia cell line, THP-1	oxLDL	Huang et al. (2013)
	*In vitro*	Berberine	Human umbilical vein endothelial cells	AMPK, eNOS, NOX4	Zhang et al. (2013)
	*In vitro*	Berberine	Human umbilical vein endothelial cell line and the human premonocytic cell line U937	Ang II	Ko et al. (2007)
	*In vitro*	Berberine	Raw 264.7 macrophages and 3T3-L1 adipocytes	AMPK	Jeong et al. (2009)
	*In vitro*	Berberine	Human umbilical vein endothelial cells	oxLDL, TNFα	Caliceti et al. (2017)
	*In vitro*	Berberine	Monocytic THP-1 cells, human monocyte line	NLRP3 and IL-1β	Liu et al. (2016)
Coronary atherosclerosis	*In vitro*	Berberine	Bone marrow cells, Human THP-1 cells, Murine 3T3L-1 cells	NLRP3 inflammasome	Zhou et al. (2017)
	clinical trials	Berberine (500 mg, daily)	Mild or moderate mixed hyperlipidemia patients	LDL and total triglycerides (TG)	Cicero et al. (2007)
	Clinical trials	Berberine capsules (900 mg/d for 3 months)	Patients with mild hyperlipidemia	Plasma total cholesterol (TC) and LDL-C	Wang et al. (2016)
	Clinical trials	Berberine nutritional agents (containing Berberis aristata d.e. 588 mg)	Patients with mild to moderate hypercholesterolemia	LDL-C	D’addato et al. (2017)
Myocardial infarction/Reperfusion	*In vivo*	Berberine	Sprague Dawley (SD) rats	Phosphoinositide 3-kinase/AKT	Qin-Wei and Yong-Guang, (2016)
	*In vivo*	Berberine	Male Wistar rats	AMPK and the AKT/GSK3b signaling pathway	Chang et al. (2016)
	*In vivo*	Berberine	Male Sprague-Dawley rats	Apoptosis and mitochondrial dysfunction	Wang et al. (2015)
	*In vivo*	Berberine	Male Sprague-Dawley rats	Silent information regulator 1 (SIRT1)	Yu et al. (2016)
	*In vivo*	Berberine	C57BL/6 mice	NF-κB and PI3K/AKT	Wang et al. (2018)
	*In vitro*	Berberine	H9C2 embryonic rat myocardium-derived cells	JAK2/STAT3	Zhao et al. (2016)
	*In vitro*	Berberine	H9C2 cardiomyocytes	Silent information regulator 1 (SIRT1)	Yu et al. (2016)
Myocardial infarction/Reperfusion	Clinical trials	Berberine (300 mg (tid) in addition to the therapy of the general group)	In acute ischemic stroke (AIS) patients	The serum macrophage migration inhibitory factor and IL-6 levels	Li Y. et al. (2016b)
	Clinical trials	Berberine tablets (0.3 g/time, and three times/day)	Patients with acute myocardial infarction treated with percutaneous coronary intervention	Plasma level of C-reactive protein, tumor necrosis factor α and IL-6	Qing et al. (2018)
Arrhythmia (atrial fibrillation)	*In vivo*	—	p47^−/−^ mice; MsrA^−/−^ mice; mice with genetic CaMKII inhibition	Oxidized CaMKII	Erickson et al. (2008), Swaminathan et al. (2011)
	*In vivo*	—	Male C57BL/6	Reactive oxygen species signaling	Yang X. et al. (2020)
	*In vitro*	—	Human Jurkat T cell line	Reactive oxygen intermediates/CaM kinases	Howe et al. (2004)
Heart failure	*In vivo*	—	Nampt transgenic mice	NAD+ synthesis	Hsu et al. (2009)
	*In vivo*	—	Male NOS3-null (NOS3^−/−^) mice and C57/BL6 WT mice	151	Takimoto et al. (2005)
	*In vivo*	—	p47^phox−/−^ mice and WT mice	NAD(P)H Oxidase Subunit p47phox	Doerries et al. (2007)
	*In vivo*	—	SRF^HKO^ and control (Sf/Sf) mice	NAD+	Diguet et al. (2018)
	*In vivo*	—	Wistar Kyoto and spontaneously hypertensive/HF (SHHF) rat	Xanthine Oxidoreductase	Minhas et al. (2006)
	*In vivo*	—	Dogs	Xanthine oxidase	Ukai et al. (2001)
	*In vitro*	—	Primary cultures of cardiac myocyte	NAD+ and Sir2α deacetylase	Pillai et al. (2005)

## 2 Chemistry and Bioactivity of Berberine

**FIGURE 1:**
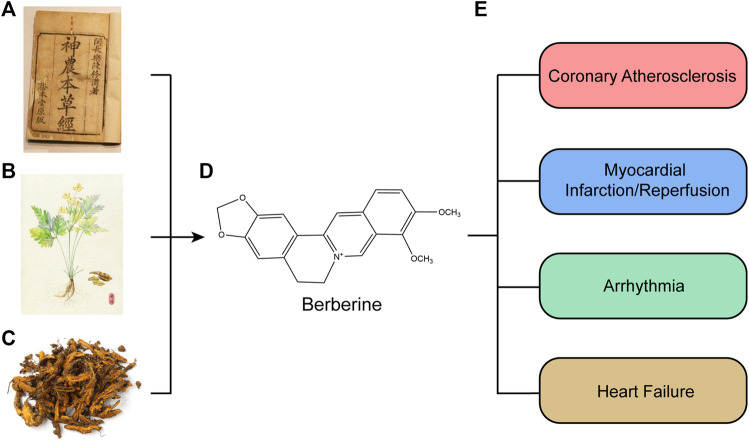
Source and functions and of berberine extract. **(A)** “Shennong’s Classic of Material Medical.” **(B)** Complete morphology of Chinese herb Huanglian. **(C)** Prepared officinal part of Chinese herb Huanglian. **(D)** Chemical structures of berberine compounds. **(E)** Pharmacological effects of berberine. Berberine was first documented in “Shennong’s Classic of Material Medical” in China. Berberine is isolated from Hydrastis canadensis, the Chinese herb Huanglian, and many other plants, such as the Berberidaceae and Ranunculaceae families. Berberine has many other potential pharmacological effects on various diseases, Furthermore, they have been known to have antiatherosclerosis, antimyocardial ischemia/reperfusion, and several other effects.

Berberine (BBR) is a natural product extracted from the roots, rhizomes, and stem bulk of the Berberidaceae and Ranunculaceae families (such as Hydrastis canadensis, the Chinese herb Huanglian, and many other plants) (Pang et al., 2015; Tan et al., 2016; Ye et al., 2017; Neag et al., 2018; Feng X. et al., 2019) (Figure 1). It has the molecular formula [C20H18NO4]^+^ and a molar weight of 336.36 g/mol (Battu et al., 2010; Tan et al., 2016). 5,6-dihydro-9,10-dimethoxybenzo [g]-1,3-benzodioxolo [5,6-á] quinolizinium is the chemical name for BBR (Lau et al., 2001). BBR is a yellow needle-shaped crystal that may be precipitated in ether and has a melting temperature of 145°C. It exhibits antimicrobial effects on hemolytic *Streptococcus*, *Staphylococcus aureus*, *Neisseria gonorrhoeae*, and Freund’s *Shigella*, and can improve leukocyte phagocytosis (Ye et al., 2017). Because of extensive antibacterial activity, extracts of BBR-containing plants or BBR have been the most successful folkloric therapy to against dysentery and infectious diarrhea in China for centuries (Lau et al., 2001; Neag et al., 2018). BBR has many other potential pharmacological effects on various diseases, it has been known to exert anti-inflammatory and anti-cancer effects (Neag et al., 2018); in addition, it can also induce anti-oxidative stress activities (Ye et al., 2017; Neag et al., 2018).

**FIGURE 2:**
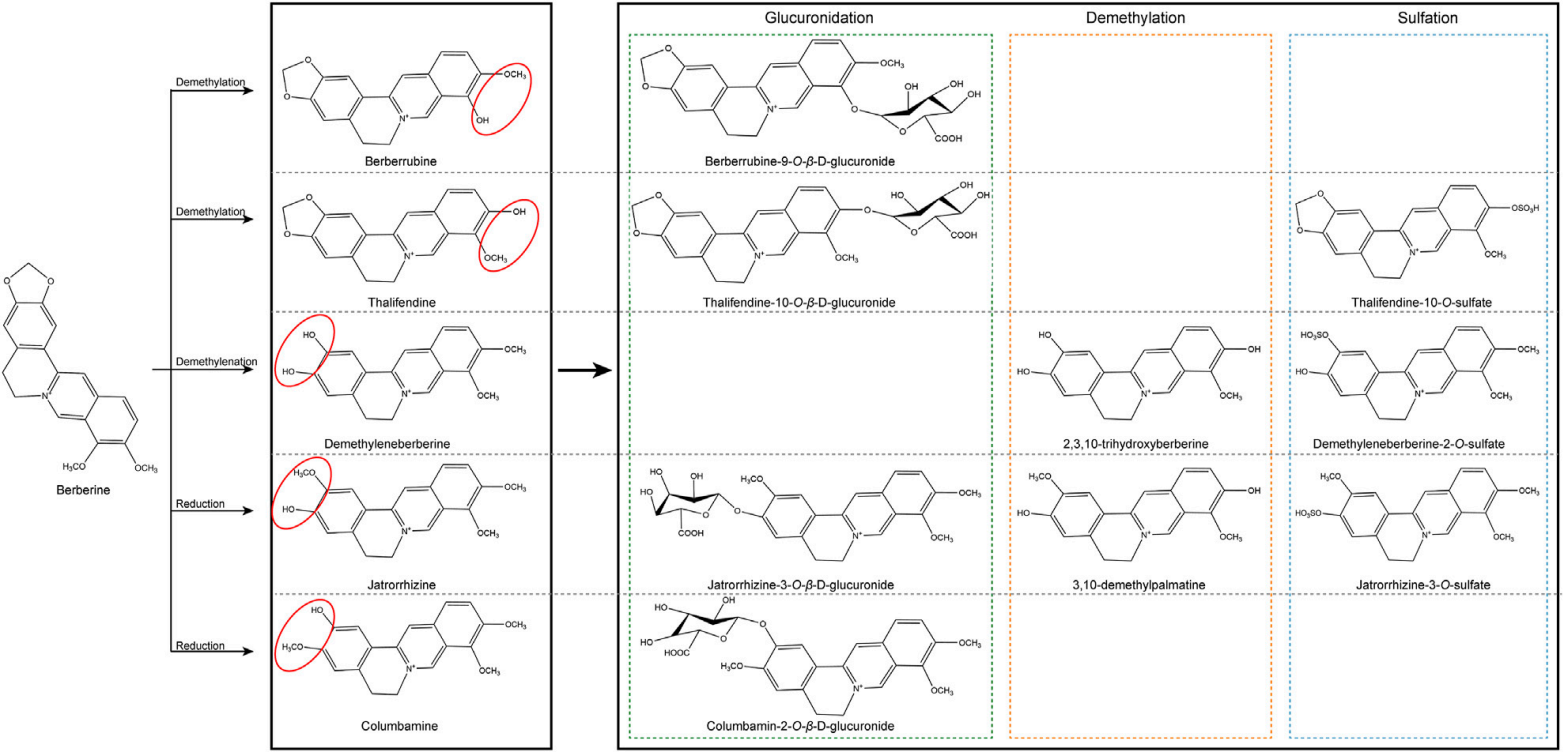
Chemical structure of active components of berberine. BBR is metabolized in the body by metabolic pathways (such as demethylation, glucuronidation etc) to thalifendin, berberrubine, jatrorrhizin, demethyleneberberin.

BBR is rapidly transformed after oral application into phase I products, which are then coupled with glucuronic acid or sulfuric acid to create phase II metabolites, which are finally discharged from urine and bile (Ma et al., 2013; Wang K. et al., 2017). The principal metabolic pathways (Figure 2) for BBR in humans and rats are demethylation, demethylenation or reduction and succedent interaction with glucuronic acid and sulfuric acid (Wang K. et al., 2017; Feng X. et al., 2019).

However, BBR itself is of low bioavailability (with a bioavailability of less than 1%), Feng W. et al. (2019), Feng et al. (2020) have described that gut microbiota metabolites of BBR could reveal the enigma between low bioavailability and powerful therapeutic effects. In gastrointestinal tract, nitroreductases are bacterial enzymes that could catalyze the reductive reaction of BBR (Li Y. H. et al., 2009). Nitroreductases regulates BBR absorption by converting BBR into absorbable dihydroberberine, which is 5–10 times more absorbed than BBR (Li Y. H. et al., 2009). After oral administration of BBR, the bioavailability of BBR was significantly increased in hamsters fed a high-fat diet compared with hamsters fed a normal diet (Li Y. H. et al., 2009). Interestingly, BBR can reduce blood lipids in high-fat hamsters, but it has no obvious lipid-lowering effect on normal hamsters. The interaction of nitroreductases with BBR in the gut has also been demonstrated in rats (Feng et al., 2015). Clinical studies have shown that a positive relationship between blood concentration of BBR and activity of fecal nitroreductase (Li Y. H. et al., 2009; Wang Y. et al., 2017). There is a study showed that BBR could modulate branched-chain amino acids biosynthesis, degradation, and transport in gut microbiota, which alleviate insulin resistance in animals (Yue et al., 2019). The above-mentioned results indicate that by studying the interaction between gut microbiota and BBR, we can change the predicament of BBR’s low bioavailability, which will make it better biologically.

## 3 Molecular Mechanisms of Oxidative Stress in Cardiovascular Diseases

**FIGURE 3:**
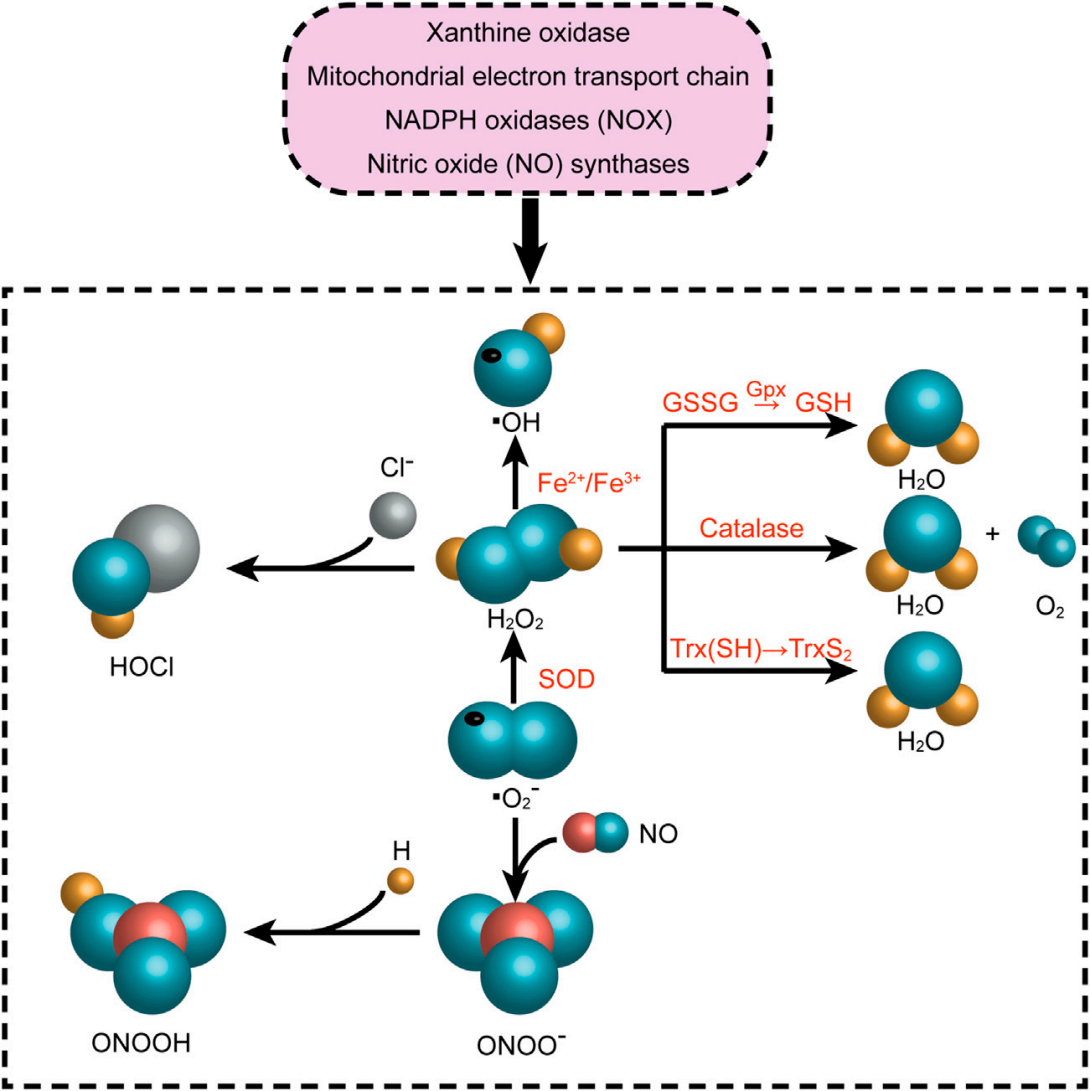
Reactive oxygen species-producing systems in cardiovascular diseases. O_2_^●−^ can be generated in extracellular myocardium by NAD(P)H, uncoupled eNOS, xanthine oxidase, and mitochondrial respiration chains. H_2_O_2_ can be spontaneously converted into OH^●−^ by Fe reaction and SOD. H_2_O_2_ can be detoxified by GSH peroxidase, Trx peroxidase, and catalase to H_2_O and O_2_. The myeloperoxidase enzyme can employ H_2_O_2_ to oxygenize chloride to the strong oxidizer HOCl. In addition, the decoupling of eNOS reduces the production of NO^●^ in endothelial cells, and the decrease in the expression and activity of eNOS further aggravates the production of NO^●^. NAD(P)H, nicotinamide adenine dinucleotide (phosphate); eNOS, endothelial nitric oxide synthase; NO^●^, nitric oxide; O_2_^●−^, superoxide; HOCl, hypochlorite; SOD, superoxide dismutase activity; H_2_O_2_, hydrogen peroxide; ONOO^−^, peroxynitrite; OH^●^, hydroxyl radicals; GSH, glutathione; GSSG, oxidized glutathione; GPx, glutathione peroxidase; Trx, thioredoxin.

Reactive oxygen species (ROS) play a significant part in many CVDs, and their unregulated generation is related to myocardial tissue damage. The state of oxidative stress results from an imbalance in the formation of ROS and antioxidant defenses in the body, which resulting in an accumulation of ROS and eventually damage cells and tissues (Pizzino et al., 2017; Charlton et al., 2020). In physiological situations, appropriate amount of ROS exerts an influence on cellular signal transduction and physiological function, which is equivalent to their detoxification effect (Tsutsui et al., 2009).It is known as the redox signaling characterized by specific and invertible oxidation/reduction modification of cell signal elements that can be modulated, and can affect gene expression, excitation-contraction coupling, or cell growth, migration, differentiation, and death (Burgoyne et al., 2012; Sack et al., 2017). The process of ROS production mainly includes enzymatic or non-enzymatic reactions, which are generated by oxidase and then eliminated by the scavenging system. The imbalance between ROS generation and removal systems results in increase in ROS levels and represent alarming stresses. Almost every subcellular organelle in the cell produces ROS (Corpas et al., 2015; Sun et al., 2020). At the cardiac level, the mitochondrial electron transport chain, xanthine oxidase, nicotinamide adenine dinucleotide phosphate (NADPH) oxidase (NOX) and nitric oxide (NO) synthases are the primary producers of ROS (Figure 3). In our prior investigation, we discovered that the activity of ROS was increased by ibrutinib in mice, which boosted the synthesis of atrial fibrillation (AF) maintenance substrates, and ultimately making them more susceptible to AF. Apocynin is a NOX inhibitor that can reduce the occurrence of AF and atrial remodeling (Yang X. et al., 2020). On the one side, NOXs (summarized as the NOX enzyme family) are the only major ROS resources (Drummond et al., 2011; Lassegue et al., 2012; Xu T. et al., 2019). There is ample evidence that NOX enzymes play a key role in the pathophysiology of several CVDs (Brandes et al., 2010; Lassegue et al., 2012; Senoner and Dichtl, 2019). On the other side, the activity of the mitochondrial electron transport chain (ETC) generates ATP to meet cellular energy requirements. In most cell types, oxygen acts as an electron acceptor, which is another ROS resource (Murphy, 2009; Xu T. et al., 2019). In addition, many other enzymes such as xanthine oxidase, nitric oxide synthase, cyclooxygenase, cytochrome P450 enzymes and lipoxygenase, as well as other organelles, such as peroxisomes and endoplasmic reticulum, can promote the production of ROS in cells (Senoner and Dichtl, 2019). Intracellular ROS is strongly linked to the pathogenesis of CVDs, such as atherosclerosis (AS), myocardial ischemia/reperfusion (I/R) injury, arrhythmia, and HF (Xu T. et al., 2019; Sun et al., 2020).

## 4 Effect of Berberine on Cardiovascular Diseases

### 4.1 Coronary Atherosclerosis

AS is currently considered a chronic inflammatory illness. Hyperactivated pro-inflammatory signaling pathways, increased oxidative stress and upregulated cytokines/chemokines expression are crucial factors in the pathogenesis of AS. The excessive production of ROS causes oxidative stress, which has become an essential and final common mechanism of AS (Kattoor et al., 2017). Some researches on mouse models of AS suggest that BBR may have an anti-atherosclerotic effect (Wu et al., 2020; Yang X.-J. et al., 2020). BBR plays a protective and ameliorative role in AS by regulating various cellular events that pro-atherogenic, including the reduction of oxidative stress (Cheng et al., 2013).

#### 4.1.1 *In Vivo*

Endothelial dysfunction and cell damage is caused by oxidative stress, which plays a key role in the onset and progression of cardiovascular disorders, such as AS (Xi et al., 2007). In the ApoE^−/−^ mouse model, BBR, particularly 8-BBR-C16 administration, effectively inhibited nuclear factor kappa-B (NF-κB) activation and reduced the protein expression of ICAM-1, iNOS and interleukin (IL)-6. These results indicate that the inhibition of BBR and 8-BBR-C16 on the development of AS in ApoE^−/−^ mice can be achieved not only by reducing lipids levels, but also by enhancing anti-inflammatory and antioxidant capabilities (Feng et al., 2017). BBR is a protective agent for endothelial cells. There is a study has found that BBR improves the stability of atherosclerotic plaques in mice with hyperhomocysteinemia, which is associated with the stimulation of peroxisome proliferator-activated receptor-c and consequent reduction of oxidative stress in endothelial cells (Li H. et al., 2016). BBR treatment can inhibit endoplasmic reticulum stress and thereby eliminate ROS, resulting in cyclooxygenase (COX)-2 down-regulation (Liu et al., 2015) or reduction of endothelial microparticle-mediated oxidative stress, thereby improving endothelial function (Cheng et al., 2013). It has been demonstrated that probiotics can influence the lipid metabolism and effectively reduce AS by means of regulating gut microbiota to decrease trimethylamine oxide (Liang et al., 2020). The study has shown that different short chain fatty acids have different effects on the activation of Nod-like receptor family pyrin domain containing-3 (NLRP3) and formation of arterial neointima. Among short chain fatty acids, butyrate has promising therapeutic effects and can serve as a new source of therapeutic strategies for AS (Yuan et al., 2018). An article concluded that the regulatory role of BBR in metabolic abnormalities and AS is achieved through the interaction with the gut microbiota (Cao et al., 2021). The research indicated that in high-fat diet-fed rats, BBR restored the gut barrier, decreased inflammatory and oxidative stress markers, and improved gut peptide levels via regulating gut microbiota (Xu et al., 2017). In ApoE^-/-^ mice fed a high-fat diet, BBR can improve serum lipid and systemic inflammation levels, and alleviate AS, which may be partly due to changes in composition and functions of gut microbiota (Wu et al., 2020). A similar animal study showed that BBR alleviated AS development and decreased inflammatory cytokine expression, which is related to alterations in gut microbiota compositions (Shi et al., 2018). Gut microbiota are of great value in the treatment of disease processes, and studies have found that modulating gut microbiota, especially increasing the abundance of Akkermansia, may be helpful to improve the anti-atherosclerotic and metabolic protective effects of BBR (Zhu et al., 2018).

#### 4.1.2 *In Vitro*

Atherosclerosis is an inflammation-driven disease and macrophages have a central role in the modulation of inflammation. Two major macrophages phenotypes have been characterized according to the activation by different microenvironmental signals: pro-inflammatory M1 macrophages induced by bacterial lipopolysaccharide (LPS) and/or interferon gamma (IFN-γ) and anti-inflammatory M2 macrophages triggered by IL-4 or IL-13 (Kuznetsova et al., 2020; Yang et al., 2021). There is research suggested that BBR not only inhibit M1 macrophages polarization, but also promote M2 macrophages polarization (Yang et al., 2021). Activated macrophages produce a number of inflammation-related factors such as IL-1β, TNF-α, IL-6, IL-8, matrix metalloprotease-9 (MMP-9), and so on, which initiate inflammation to induce AS (Kleemann et al., 2008). Some studies suggest that BBR significantly downregulated the proinflammatory cytokines (TNF-α, IL-6, IL-1β, and MCP-1) (Jeong et al., 2009; Wang et al., 2020). NOX may be the most important ROS generation system in the cardiovascular system (Kattoor et al., 2017). BBR inhibited the generation of ROS, which may be caused by the inhibition of the activated NOX content (Jiang et al., 2017). On the one hand, NOX activity in macrophages is an important part of the production of oxidized low-density lipoprotein (ox-LDL) (Kattoor et al., 2017). Ox-LDL, which stimulates circulating monocytes and increases their potential to penetrate blood vessel walls, is one of the novel and high-profile cardiovascular risk factors. Increased infiltration is a critical factor in the occurrence of AS (Huang et al., 1999; Cipolletta et al., 2005; Hulsmans and Holvoet, 2010). BBR can stimulate macrophage autophagy through AMPK/mTOR signaling pathway, thereby attenuating the inflammatory markers generated by ox-LDL (Fan et al., 2015). In human umbilical vein endothelial cells (HUVECs), oxidative stress is alleviated to a sufficient degree to normalize ApoB fragmentation after BBR treatment. It is suggested that BBR has strong antioxidant capacity and inhibits LDL oxidation (Hsieh et al., 2007). BBR significantly reduced the adhesion of monocytes and HUVECs induced by ox-LDL, and this effect was positively correlated with the dose. The experiments show that BBR has a protective effect in the early stage of AS (Huang et al., 2013). On the other hand, the activation of NOX can promote the formation of ROS, leading to endothelial dysfunction (Sena et al., 2013). NOX4 is a subtype of NOX, which is mainly expressed in vascular endothelial cells and is the main source of endothelial cells to produce O_2_^−^ (Zhang et al., 2013). In palmitic acid-treated HUVECs, BBR reduced ROS production and NOX4 protein expression (Zhang et al., 2013). Moreover, excessive ROS, up-regulated monocyte chemoattractant protein (MCP)-1, and increased adhesion of monocytes to endothelial cells induced by Ang II can all be effectively inhibited by BBR (Ko et al., 2007). According to research findings, BBR mainly inhibits the accumulation of ROS derived from NOX4 in HUVECs (Zhang et al., 2013) or the production of cellular ROS in macrophages by activating AMPK (Jeong et al., 2009). BBR treatment can reduce the level of ROS in endothelial cells after acute exposure to H_2_O_2_ (Caliceti et al., 2017). Furthermore, the activation of NLRP3 inflammasomes can also be suppressed by BBR: BBR reduces the activation of ROS-dependent NLRP3 inflammasomes in macrophages and inhibits NF-κB, which inhibits the expression and release of IL-1β (Jiang et al., 2017; An et al., 2019). BBR reduces the activation of inflammasomes induced by natriuretic acid crystals by inhibiting the expression of NLRP3 and IL-1β (Liu et al., 2016). Furthermore, BBR also inhibits the action of NLRP3 inflammasomes and the production of IL-1β induced by saturated fatty acids (palmitate) in adipose tissue-derived macrophages through activating AMPK-dependent autophagy (Zhou et al., 2017).

#### 4.1.3 Clinical Trials

The lipids that cause AS, particularly low-density lipoprotein cholesterol (LDL-C), have occasional effects on the occurrence and development of atherosclerotic plaques (Ference et al., 2017; Fatahian et al., 2020). Numerous clinical trials have been conducted to investigate the role of BBR in the treatment of atherosclerotic lipids. It has been shown that in mild or moderate mixed hyperlipidemia patients, 500mg of BBR daily were able to significantly reduce LDL and total triglycerides (TG). And no adverse events were reported during the study (Cicero et al., 2007). A clinical randomized controlled trial concluded that oral BBR capsules (900 mg/d for 3 months) were effective in reducing plasma total cholesterol (TC) and LDL-C levels compared with placebo in patients with mild hyperlipidemia (Wang et al., 2016). A multicenter, randomized, double-blind, placebo, controlled trial confirmed the safety and efficacy of BBR in patients with mild to moderate hypercholesterolemia, where continuous daily oral BBR nutritional agents (red yeast rice, coenzyme Q10, and hydroxytyrosine, containing Berberis aristata d.e. 588 mg) for 4 weeks induced a 26% reduction in LDL-C (D’addato et al., 2017). The study suggests that combined administration of monacolin K and BBR could provide similar protection from CVDs compared to prescription statin therapy, with potentially lower risks for adverse effects (D’addato et al., 2017). In addition, there are some meta-analyses showed that BBR was successful in reducing TC and LDL cholesterol. A meta-analysis that included 19 controlled and cross-sectional trials indicated that the combination of BBR with silymarin significantly lowered cholesterol (Bertuccioli et al., 2020). Moreover, a meta-analysis including 16 randomized controlled trials with 2,147 participants strongly confirmed the BBR’s efficacy and safety in the treatment of dyslipidemia patients. They thought that the blood lipid status of patients with dyslipidemia was significantly improved after BBR treatment, and the safety was satisfactory, the contents of TC and LDL-C were significantly decreased, and no significant adverse events occurred (Ju et al., 2018). Another clinical study and meta-analysis showed that BBR combined with nutritional supplements or combined oral administration of BBR and drugs could reduce plasma LDL-C and TC levels without significant side effects (Affuso et al., 2010; Marazzi et al., 2011; Pisciotta et al., 2012; Cicero et al., 2019; Zhang et al., 2019). In conclusion, these studies all confirm that BBR regulates plasma cholesterol levels in patients with dyslipidemia and that this natural compound provides an exciting and encouraging preventive and therapeutic strategy for AS.

### 4.2 Myocardial Infarction/Reperfusion

Myocardial infarction is one of the common ischemic heart diseases and is the main cause of mortality in high-income societies (Kim et al., 2009). Reperfusion itself can lead to other injuries, such as cardiac dysfunction, reperfusion arrhythmia, and aggravated myocardial infarction. ROS is critical for organ damage during I/R (Cross et al., 1987; Halliwell et al., 1992; Jeroudi et al., 1994). Numerous researches have demonstrated that the formation of ROS increases regardless of whether it is during I/R (Minutoli et al., 2016). The mechanisms by which excessive ROS induce cellular injury involve interference with cell signal transduction, activation of inflammatory factors, lipid peroxidation (Davidson et al., 2012) and even direct cell death (Zhan et al., 2016). BBR has antioxidant and anti-inflammatory effects, meanwhile, BBR treatment has a cardioprotective effect on I/R injury, and can significantly improve cardiac function after I/R injury (Qi et al., 2010; Chang et al., 2012; Gao et al., 2012; Chang et al., 2016; Zhao et al., 2016).

#### 4.2.1 *In Vivo*

There is a study offered direct evidence that BBR-treated rats played an antioxidant role by reducing cardiac superoxide production, gp91phox expression, malondialdehyde (MDA) concentration and promoting superoxide dismutase (SOD) activity (Yu et al., 2016). In previous research, the levels of MDA, a marker of lipid peroxidation (Chang et al., 2016), were significantly reduced in the BBR-treated diabetic rat model (Chang et al., 2016). Sirtuin 1 (SIRT1)-mediated antioxidant effects have been shown to exert a protective effect on I/R myocardium (Zhang et al., 2018). Mitochondria is the center of energy production. Ischemia triggers energy depletion, leading to mitochondrial dysfunction, which increases oxidative stress and induce apoptosis (Bulteau et al., 2005; Bhatti et al., 2017). In turn, oxidative stress aggravates ischemia reperfusion injury (Bulteau et al., 2005; Wang et al., 2015; Bhatti et al., 2017). In myocardial ischemia-reperfusion model of male Sprague-Dawley rats, the mitochondrial membrane potential (MMP) is improved due to BBR pretreatment, indicating that I/R-induced mitochondrial lesion was obviously reduced (Wang et al., 2015). Yu et al. (2016) found that SIRT1 inhibitors abrogated the antioxidant effect of BBR using a rat myocardial I/R injury model, suggesting an indispensable role of SIRT1 in the cardioprotective effect of BBR. The anti-inflammatory effect of BBR is associated with its inhibition of phosphoinositide 3-kinase (PI3K)/AKT signaling pathway, which reduces the secretion of a variety of pro-inflammatory cytokines/mediators in cardiac myocytes and serum, such as IL-6, IL-1β and tumor necrosis factor (TNF)-α in serum and cardiac myocytes (Shang et al., 2010; Li et al., 2014; Qin-Wei and Yong-Guang, 2016). In addition, BBR also alleviated inflammation by suppressing the NF-κB signaling pathway (Wang et al., 2018). Moreover, it has been reported that the BBR derivative rainanberine protects pulmonary arterial ring and by inhibiting NOX and calcium influx (Gao et al., 2012). In addition, Liu et al. (2017) suggest a potential new strategy by which exercise benefits the cardiovascular system by altering the microbiome. Zununi Vahed et al. (2018) reviewed the development of microbiota dysbiosis after myocardial infarction and gave recent advances in a microbiota-based therapeutic strategy to delay or prevent myocardial infarction. Administration of antibiotics decrease or increase the abundance of specific bacterial groups in the rat intestine, which links to severity of myocardial infarction and may provide opportunities for novel diagnostic tests and interventions for prevention of CVD (Lam et al., 2012; Lam et al., 2016). Beneficial microbiota reduce MI risk factors or reduce post-MI events mainly by modulating lipogenesis and cholesterol metabolism and antioxidant production (Girard et al., 2009; Lam et al., 2012; Mccafferty et al., 2012; Gan et al., 2014). As a consequence, further studies are needed to devise the impact of BBR on intestinal microbiota and myocardial infarction though ROS.

#### 4.2.2 *In Vitro*

In addition, the antioxidant effect of BBR was also observed in cultured cells (Tan et al., 2007; Chatuphonprasert et al., 2013). BBR significantly attenuated I/R injury in SIR-injured H9C2 cardiomyocytes, and the SIRT1 signaling pathway more or less mediated this cardioprotective property characterized by antioxidant and anti-inflammatory effects (Yu et al., 2016). Furthermore, SIR-induced cardiac apoptosis, oxidative stress and endoplasmic membrane stress were remarkably downregulated by BBR (Zhao et al., 2016).

#### 4.2.3 Clinical Trials

Unfortunately, although a large number of preclinical studies have conclusively concluded that BBR has a preventive and curative effect on I/R, its clinical studies are scarce (Liu et al., 2019). For the first time, Li Y. et al. (2016) found evidence that neurological deficits and the prognosis were improved in acute ischemic stroke (AIS) patients. The BBR group received BBR 300 mg (tid) in addition to the therapy of the general group. They believe that BBR worked by downregulating the serum macrophage migration inhibitory factor and IL-6 levels. And there was no significant difference in the incidence of adverse reactions between the two groups (Li Y. et al., 2016). Similarly, the BBR group was given additional BBR tablets 0.3 g/time, and taken three times a day. The results suggest that BBR prolonged the life expectancy and improved the quality of life in patients with acute myocardial infarction treated with percutaneous coronary intervention (Qing et al., 2018). Moreover, they also discovered that the plasma level of C-reactive protein, tumor necrosis factor α and IL-6 were considerably decreased by BBR treatment. In summary, all of these findings indicated that BBR may alleviate I/R injury by inhibiting the over-reactive inflammatory response.

### 4.3 Arrhythmia

Arrhythmia is defined as an abnormality of the heart rate or rhythm due to an abnormality in the frequency, rhythm, pacing site, conduction velocity, or sequence of excitation of the heart impulses, with AF being the most prevalent and closely linked to high cardiovascular morbidity and mortality (Nattel and Dobrev, 2016; Barangi et al., 2018).

#### 4.3.1 Oxidative Stress in Atrial Fibrillation

Atrial structural remodeling, electrical remodeling, alterations in the autonomic nervous system, and Ca^2+^ disturbances are the four major mechanisms of AF pathogenesis. In addition, increased ROS might alter ion channel activity, thereby increasing AF susceptibility (Barangi et al., 2018). ROS via modifying proteins central to excitation–contraction coupling, including L-type calcium channels, sodium channels, potassium channels, and sodium-calcium exchangers, which may contribute to the pathogenesis of the arrhythmia (Takimoto and Kass, 2007). ROS also affects the function of the sarcoplasmic reticulum Ca^2+^-adenosine triphosphatase (SERCA) and changes the activity of calcium sensitivity in myofilaments. Additionally, ROS causes an energy deficit by interfering with the action of energy-metabolizing proteins. Eventually, ROS promotes fibrosis via increasing cardiac fibroblast proliferation and matrix metalloproteinases, which results in extracellular remodeling (Takimoto and Kass, 2007; Van Der Pol et al., 2019). On the one hand, excessive ROS was detected in cardiac tissue of several atrial pacing models, which leads to electrical changes in the atria and ultimately induces AF (Violi et al., 2014; Karam et al., 2017; Korantzopoulos et al., 2018). On the other hand, the role of oxidative stress in the pathogenesis of AF has been increasingly recognized. The risk factors that induce AF, such as adiposity, diabetes, age, and hypertension, may all be linked by oxidative stress (Ziolo and Mohler, 2015). Accelerated ROS in myocardial tissues causes DNA, protein, and lipid damage, as well as tissue injury. These events lead to heart structural and electrical remodeling, which increases susceptibility to AF (Barangi et al., 2018). Meanwhile, the multifunctional calcium/calmodulin-dependent protein kinase II (CaMKII) as a sensor is activated after being stimulated by ROS, thereby promoting the occurrence of arrhythmia (Erickson et al., 2008; Swaminathan et al., 2011). Ox-CaMKII could be regarded as a biomarker activated by ROS, mediating the development of AF (Howe et al., 2004; Erickson et al., 2008; Palomeque et al., 2008). In our previous study, we have proved the effects of ROS on atrial cardiomyocytes of mice, and it is possible that ROS activates ox-CaMKII and p-CaMKII (Thr-286) to increase AF susceptibility after ibrutinib treatment (Yang X. et al., 2020). Gut microbe-derived metabolites function primarily to modulate energy metabolism, local and systemic immune systems, and neural activity (Liu et al., 2022). Based on a strategy of metagenomic and metabolomic analyses, the study shown that the disordered gut microbiota and microbial metabolite profiles in AF. Imbalances in gut microbial function and associated changes in metabolic patterns were observed in both feces and serum of patients with AF (Zuo et al., 2019). The heart-gut axis is a potential target for CVDs therapy. A high-fructose diet can induce inflammation of the heart-gut axis and metabolic disturbances that ultimately lead to arrhythmias (Cheng W. L. et al., 2021). The current study demonstrates that aged-associated microbiota dysbiosis promotes AF in part through a microbiota-gut-atria axis. It is indicating that in fecal microbiota transplantation rat model, the microbiota-intestinal barrier-atrial NLRP3 inflammasome axis may be a reasonable molecular target for the treatment of age-related arrhythmias (Zhang et al., 2021).

#### 4.3.2 Berberine in Atrial Fibrillation

Huang et al. described the anti-arrhythmic activity of BBR for the first time in 1989 (Feng X. et al., 2019). BBR exerts its antiarrhythmic effect by prolonging action potential duration (APD), which is due to the mechanism that BBR blocks ion currents (Barangi et al., 2018). In cardiac myocytes, BBR significantly shortened the extended QTc interval while also stabilized the decreased transient outward potassium current (I_to)_ and L-type Ca^2+^ (I_Ca_) currents (Wang et al., 2012) or depressed ATP-sensitive K^+^ channel (K_ATP_) channel activation (Wang et al., 1996). The effects of BBR on cell membrane ion currents was related to its concentration, with BBR at concentrations of 0.3–30 µM blocking rapid (I_Kr_) delayed rectifier K^+^ channels and at higher dose inhibiting I_to_ (Sanchez-Chapula, 1996). Further studies revealed that BBR as well as the derivatives of BBR targets a variety of channels including the cardiac slow (I_Ks_) delayed rectifier K^+^ channels and I_Kr_, K_ATP_, inwardly-rectifying K^+^ channel (I_Kl_), I_Ca_ (Chi et al., 1996; Chi et al., 1997; Lau et al., 2001; Li et al., 2008). Na channel activity plays a major role in mediating proper action potential conduction across the heart (Karam et al., 2017). Studies have shown that elevated intracellular NADH causes a decrease in cardiac Na^+^ current (I_Na_) signaled by an increase in mitochondrial ROS (Liu et al., 2009; Liu et al., 2010). Liu et al. (2013) demonstrated a reduction in mitochondrial ROS in cardiomyopathy will reverse the reduced I_Na_ and possibly some of the arrhythmic risk by improving conduction velocity. BBR increases myocardial contractility and cardiac output via blockade of K^+^ channels, stimulation of Na^+^–Ca^2+^ exchanger, and elevation of coronary blood flow. It is considered that a transient inwardcurrent carried primarily by Na^+^, which is intimately associated with an increase in intracellular Ca^2+^ overload, is responsible for the delayed afterdepolarization. This effect is also likely to be involved in the antiarrhythmic effect of BBR (Lau et al., 2001). Recently, there are some studies that have shown that, through its favourable antioxidant and sodium channel inhibitory effects, Cannabidiol, is the main non-psychotropic constituent of the Cannabis sativa plant, may protect against high glucose-induced arrhythmia and cytotoxicity (Fouda et al., 2020; Fouda and Ruben, 2021). Future studies may reveal the antiarrhythmic effect of BBR through ROS and sodium channels. In rabbit atrial myocytes, Acetylcholine-induced AF can be inhibited by the effects of BBR to prolong APD and increase the effective refractory period of the atrium. Notably, The RR interval and effective refractory period (ERP) were likewise prolonged by BBR. According to the above-mentioned mechanisms, BBR treatment results in the termination of the acetylcholine-induced AF (Zhou et al., 2015). The IC_50_ value indicates the concentration of the inhibitor which is required to inhibit a given biological or biochemical function by half (Caldwell et al., 2012). The research found that the drug at concentrations of 0.3–30 μM blocked only the delayed rectifier (I_K_) current with an IC_50_ = 4.1 μM. BBR produced a tonic block and a phasic block that was increased with the duration of the depolarizing pulse (Sanchez-Chapula, 1996). CPU86017 is a novel Class III antiarrhythmic agent derived from BBR. It blocks I_Kr.tail_, I_Ks_, and I_Ca_ currents with IC50 values of 25, 14.4, and 11.5 μM, respectively (Dai, 2006).

Although several experiments have shown that BBR has an anti-arrhythmic impact by changing ion channel activity. Few researches have focused on the antioxidative effects of BBR in AF condition. Therefore, further research *in vivo* and vitro or clinical trials investigating the antiarrhythmic effect of BBR via ROS are warranted.

### 4.4 Heart Failure

HF is a complicated clinical syndrome caused by abnormalities in the cardiac structure or function, and its pathogenesis involves structural changes, neurohumoral, cells, and molecules (Van Der Meer et al., 2019). HF is an increasing global burden of disease characterized by altered excitation-contraction coupling, cardiac energy deficit, and oxidative stress (Weissman and Maack, 2021).

#### 4.4.1 Oxidative Stress in HF

Nicotinamide adenine dinucleotide (NAD+) and its reduced dinucleotide NADH play a pivotal role in driving oxidation-reduction reactions refer to energy production (Mericskay, 2016; Hershberger et al., 2017). In addition to its function in regulating the energy metabolism of cardiomyocytes, NAD+, as a precursor for the phosphorylated dinucleotide pair NADP+/NADPH, is vital in the detoxification of ROS (Mericskay, 2016; Hershberger et al., 2017). Reduced myocardial NAD+ levels have been observed in HF murine models (Pillai et al., 2005; Hsu et al., 2009; Rajamohan et al., 2009). Excessive ROS triggered left ventricular (LV) dilatation, LV remodeling, and consequently LV systolic dysfunction in mice (Takimoto et al., 2005). In mice myocardial infarction models, NOX inhibition lacking the cytoplasmic NOX component p47phox attenuates ventricular remodeling and dysfunction (Doerries et al., 2007). In a murine HF model, cardiac function and redox status could be improved by supplementing NAD+ levels and nicotinamide riboside (NR), a precursor of NAD+ (Diguet et al., 2018). After inhibition of xanthine oxidase using oxypurinol (rats) or allopurinol (dogs), the heart from LV remodeling was protected, the LV contractile function and myocardial efficiency post-cardiac injury was improved. These observations suggest that xanthine oxidase inhibition restores cardiac structure and function, and may enhance myocardial calcium sensitivity in HF (Ukai et al., 2001; Minhas et al., 2006). The gut microbial modulation of inflammation through short-chain fatty acids production, which is important in disease states. In patients with HF, the abundance of Ruminococcaceae on the family level was decreased and abundance of Blautia from the Lachnospiracea family on the genus level was reduced (Kamo et al., 2017), and the levels of Faecalibacterium prausnitzii was reduced (Cui et al., 2018). In HF, most of the microbes that were reduced belonged to the Lachnospiracea family, in addition to Faecalibacterium from the Ruminococcacea family (Kummen et al., 2018). Butyrate exerts local anti-inflammatory effects in the gut mucosa, and stimulates surrounding regulatory T cells (Arpaia et al., 2013). Kummen et al. (2018) reported that patients with chronic HF have reduced butyrate-producing potential bacteria (Kummen et al., 2018). Evidence suggests that elevated bacterial translocation during HF is the result of one or more mechanisms, including altered gastrointestinal structure and function during visceral hyperemia, and abnormal host immune defenses (Tang et al., 2019). There are two main ways in which gut microbiota directly interact with BBR: 1) BBR modulates gut microbiota; 2) gut microbiota transforms BBR. Researching interactions between BBR and gut microbiota will be providing reference for clinical rational use of BBR in the treatment of diseases (Cheng H. et al., 2021).

#### 4.4.2 Berberine in HF

Cardiac function in transverse aortic constriction (TAC) surgery induced chronic HF in mouse model can be remarkably ameliorated by BBR (Abudureyimu et al., 2020). In a high-dose isoprenaline (s.c.)-induced HF model in rats, 12 days of continuous administration of total saponins of Panax ginseng (20 mg/kg/d) combined with BBR (20 mg/kg/d) had similar therapeutic effects compared with captopril alone (Li Y. et al., 2009). BBR (63 mg/kg/d, p.o., 4 week) was further proven to be a promising medicine to ameliorate HF by targeting the inhibition of cardiomyocyte Ca^2+^ overload (Zhang et al., 2008). Similarly, intravenous BBR administration reduced left ventricular end-diastolic pressure and systemic vascular resistance, thereby improving cardiac output in dogs with ischemic HF (Huang et al., 1992). This activity has also been validated in other animal models (Ko et al., 2000). A randomized, double-blind controlled study concluded that BBR significantly improved left ventricular ejection fraction (LVEF), exercise capacity, dyspnea-fatigue index, and lowered the frequency and complexity of ventricular premature complexes (VPCs) in patients with chronic HF (Zeng et al., 2003). However, despite the above mentioned abundant preclinical studies suggesting an important role for oxidative stress in the development of HF, unfortunately, there is currently a lack of clinically recognized treatments that directly target ROS (Weissman and Maack, 2021). This may be due to that in the experimental setting, the most of the researches to test the efficacy of anti-oxidative stress treatments in HF models, while in the clinical setting the treatments of anti-oxidative stress were primarily tested in patients with acute myocardial infarction and not HF (Van Der Pol et al., 2019). Hypertension is a major risk factor for CVDs, including coronary artery disease, stroke, HF, AF and so on (Yousefian et al., 2019). Several studies have demonstrated the therapeutic potential of BBR and its derivatives for hypertension (Liu et al., 1999; Zhang et al., 2005; Lan et al., 2015). A systematic review investigate BBR effect on blood pressure and CVD risk in five randomized controlled trials and two non-randomized controlled trials were included with 614 participants (Suadoni and Atherton, 2021). It has been reported that natural compounds can improve hypertension due to the formation of stable free radicals with ROS-derived NADPH oxidase and prevent the assembly of NOX subunits (Yousefian et al., 2019). It was showed that NOX4-derived ROS play an important role in endothelial microparticles-induced oxidative stress, and BBR can reverse cell damage caused by elevated endothelial microparticles (Cheng et al., 2013). Tian et al. (2019) suggest that in 2K1C renovascular hypertensive rats, BBR attenuates hypertension and sympathoexcitation though the ROS/Erk1/2/iNOS pathway. Therefore, more work should be taken to investigate the mechanisms of BBR or other clinical therapies on HF via ROS and find new targets for the clinical management.

## 5 Conclusion

**FIGURE 4:**
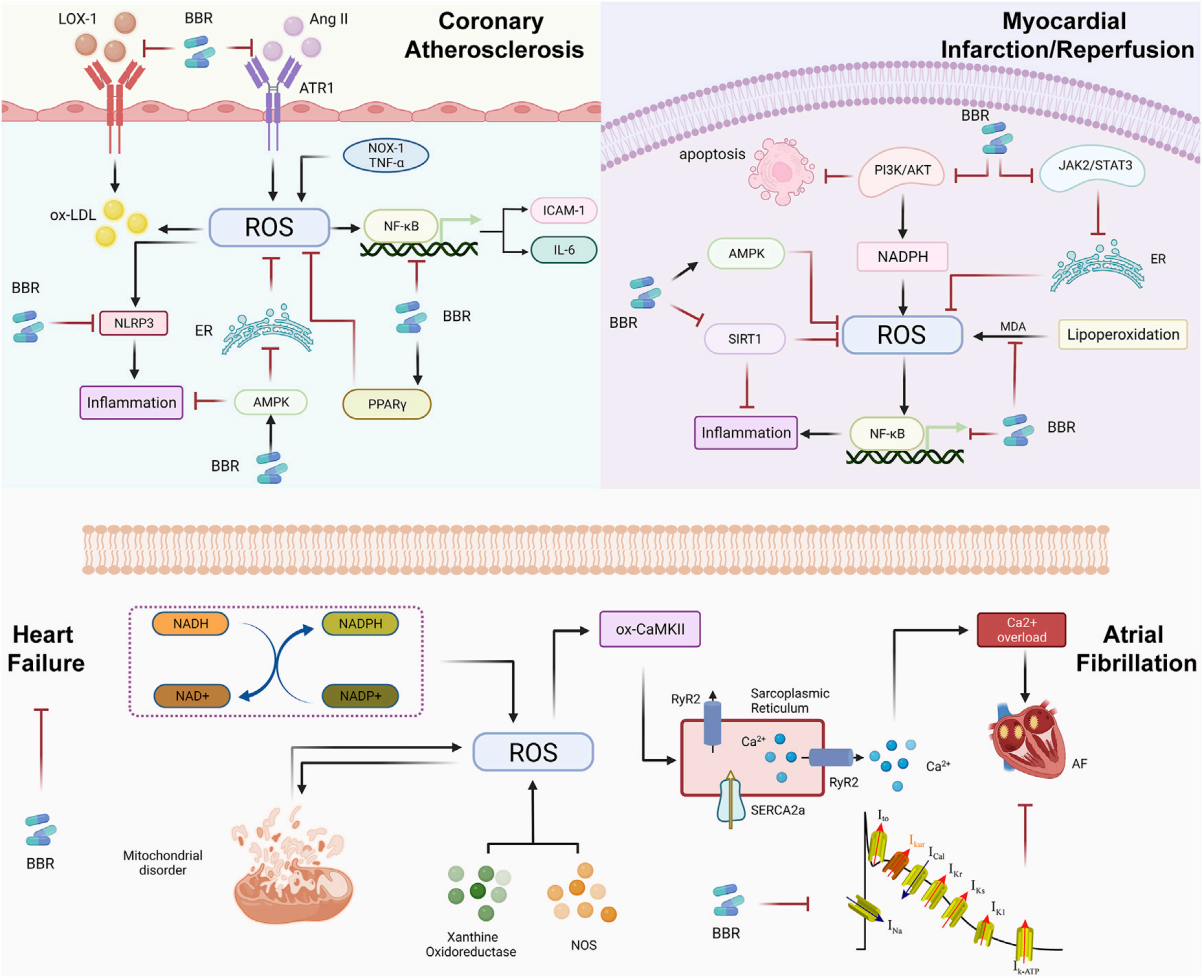
Reactive oxygen species in the evolution of cardiovascular diseases (CVDs) and pharmacological mechanism of berberine (BBR). During the evolution of atherosclerotic plaques, the main role of berberine is to inhibit the oxidation of LDL, Ang II and inflammation, or activate AMPK and PPARγ, and further inhibiting the oxidative stress response; BBR in MI/R regulates superoxide reaction by MDA, thereby preventing excessive myocardial injury. BBR may regulate oxidative stress through the NF-κB signaling, PI3K/AKT signaling, JAK2/STAT3 signaling and so on. Oxidative stress mainly affects SR and RyR2 through ox-CaMKII and causes calcium overload, which eventually leads to AF. In terms of treatment, BBR mainly reduces the occurrence of AF by inhibiting ion channels. ROS-induced HF is mainly due to NADH/NADPH, mitochondrial disorders, XO and NOS, but the improvement of berberine in HF is mostly clinical research. Therefore, it is necessary to further study the mechanisms of BBR to treat HF through ROS and find for clinical treatment new targets.

In recent years, an increasing number of studies have begun to focus on the role of BBR in CVDs. In the last decade, there are also a growing number of studies showing the unexpected therapeutic effects of BBR on many CVDs, including AS, myocardial I/R, AF and so on (Figure 4, created with BioRender.com). In this paper, we systematically and comprehensively review the chemical properties, bioavailability, and molecular mechanisms of BBR on CVDs via oxidative stress. Besides, the application of BBR in metabolic diseases is limited by its unsatisfied oral bioavailability. As a result, improving the bioavailability of BBR is a problem that needs to be addressed to expand its clinical application. It is fortunate that polymer materials and nanotechnology provide us a new idea (Zhu et al., 2013; Mirhadi et al., 2018; Xu H. Y. et al., 2019). There is research that shows that novel nanoemulsion provides a promising carrier to improve the hypoglycemic efficacy of BBR by overcoming its gastrointestinal deficiency. Nanoemulsion increased the oral bioavailability of BBR in rats by 212.02% (Xu H. Y. et al., 2019). Moreover, Mirhadi et al. (2018) describes different types of nanocarriers (polymeric based, magnetic mesoporous silica based, lipid based, dendrimer based, graphene based, silver and gold nanoparticles) have been used for encapsulation of BBR. Similarly, the self-microemulsifying drug delivery system formulation could be used as a possible alternative to traditional oral formulations of BBR to improve its bioavailability, which was enhanced about 2.42-fold compared with the commercial tablet in rats (Zhu et al., 2013). The information can be referred to for the future research related to BBR.

BBR is extensively used in clinical practice in traditional medicine. Therefore, it is worthwhile to explore the active ingredients of BBR and to elaborate the mechanism of BBR deeply from the perspectives of molecular biology and pharmacokinetics, which is beneficial to promote the broader clinical application of BBR. In short, the role of BBR in preventing and treating CVDs should not be underestimated, and rigorous, large-scale, long-term, high-quality, multicenter clinical trials need to be implemented to confirm the efficacy, safety, and economic benefits of BBR. What needs to be emphasized is that researches on the association between BBR, BBR structural analogs, BBR-containing plants and CVD, especially clinical investigations, needs to continue.

## References

[B1] Abudureyimu M. Yu W. Cao R. Y. Zhang Y. Liu H. Zheng H. (2020). Berberine Promotes Cardiac Function by Upregulating PINK1/Parkin-Mediated Mitophagy in Heart Failure. Front. Physiol. 11, 565751. 10.3389/fphys.2020.565751 33101051 PMC7546405

[B2] Affuso F. Ruvolo A. Micillo F. Saccà L. Fazio S. (2010). Effects of a Nutraceutical Combination (Berberine, Red Yeast rice and Policosanols) on Lipid Levels and Endothelial Function Randomized, Double-Blind, Placebo-Controlled Study. Nutr. Metab. Cardiovasc. Dis. 20, 656–661. 10.1016/j.numecd.2009.05.017 19699071

[B3] An N. Gao Y. Si Z. Zhang H. Wang L. Tian C. (2019). Regulatory Mechanisms of the NLRP3 Inflammasome, a Novel Immune-Inflammatory Marker in Cardiovascular Diseases. Front. Immunol. 10, 1592. 10.3389/fimmu.2019.01592 31354731 PMC6635885

[B4] Arpaia N. Campbell C. Fan X. Dikiy S. Van Der Veeken J. Deroos P. (2013). Metabolites Produced by Commensal Bacteria Promote Peripheral Regulatory T-Cell Generation. Nature 504, 451–455. 10.1038/nature12726 24226773 PMC3869884

[B5] Barangi S. Hayes A. W. Karimi G. (2018). The More Effective Treatment of Atrial Fibrillation Applying the Natural Compounds; as NADPH Oxidase and Ion Channel Inhibitors. Crit. Rev. Food Sci. Nutr. 58, 1230–1241. 10.1080/10408398.2017.1379000 28925721

[B6] Battu S. K. Repka M. A. Maddineni S. Chittiboyina A. G. Avery M. A. Majumdar S. (2010). Physicochemical Characterization of Berberine Chloride: A Perspective in the Development of a Solution Dosage Form for Oral Delivery. AAPS PharmSciTech 11, 1466–1475. 10.1208/s12249-010-9520-y 20842541 PMC2974104

[B7] Bertuccioli A. Moricoli S. Amatori S. Rocchi M. B. L. Vici G. Sisti D. (2020). Berberine and Dyslipidemia: Different Applications and Biopharmaceutical Formulations without Statin-like Molecules-A Meta-Analysis. J. Med. Food 23, 101–113. 10.1089/jmf.2019.0088 31441678

[B8] Bhatti J. S. Bhatti G. K. Reddy P. H. (2017). Mitochondrial Dysfunction and Oxidative Stress in Metabolic Disorders - A Step towards Mitochondria Based Therapeutic Strategies. Biochim. Biophys. Acta Mol. Basis Dis. 1863, 1066–1077. 10.1016/j.bbadis.2016.11.010 27836629 PMC5423868

[B9] Brandes R. P. Weissmann N. Schröder K. (2010). NADPH Oxidases in Cardiovascular Disease. Free Radic. Biol. Med. 49, 687–706. 10.1016/j.freeradbiomed.2010.04.030 20444433

[B10] Bulteau A. L. Lundberg K. C. Ikeda-Saito M. Isaya G. Szweda L. I. (2005). Reversible Redox-dependent Modulation of Mitochondrial Aconitase and Proteolytic Activity during *In Vivo* Cardiac Ischemia/reperfusion. Proc. Natl. Acad. Sci. U.S.A. 102, 5987–5991. 10.1073/pnas.0501519102 15840721 PMC1087934

[B11] Burgoyne J. R. Mongue-Din H. Eaton P. Shah A. M. (2012). Redox Signaling in Cardiac Physiology and Pathology. Circ. Res. 111, 1091–1106. 10.1161/CIRCRESAHA.111.255216 23023511

[B12] Caldwell G. W. Yan Z. Lang W. Masucci J. A. (2012). The IC(50) Concept Revisited. Curr. Top. Med. Chem. 12, 1282–1290. 10.2174/156802612800672844 22571790

[B13] Caliceti C. Rizzo P. Ferrari R. Fortini F. Aquila G. Leoncini E. (2017). Novel Role of the Nutraceutical Bioactive Compound Berberine in Lectin-Like OxLDL Receptor 1-mediated Endothelial Dysfunction in Comparison to Lovastatin. Nutr. Metab. Cardiovasc. Dis. 27, 552–563. 10.1016/j.numecd.2017.04.002 28511903

[B14] Cao R. Y. Zhang Y. Feng Z. Liu S. Liu Y. Zheng H. (2021). The Effective Role of Natural Product Berberine in Modulating Oxidative Stress and Inflammation Related Atherosclerosis: Novel Insights into the Gut-Heart Axis Evidenced by Genetic Sequencing Analysis. Front. Pharmacol. 12, 764994. 10.3389/fphar.2021.764994 35002703 PMC8727899

[B15] Chang W. Li K. Guan F. Yao F. Yu Y. Zhang M. (2016). Berberine Pretreatment Confers Cardioprotection against Ischemia-Reperfusion Injury in a Rat Model of Type 2 Diabetes. J. Cardiovasc. Pharmacol. Ther. 21, 486–494. 10.1177/1074248415627873 26846272

[B16] Chang W. Zhang M. Li J. Meng Z. Xiao D. Wei S. (2012). Berberine Attenuates Ischemia-Reperfusion Injury via Regulation of Adenosine-5'-Monophosphate Kinase Activity in Both Non-ischemic and Ischemic Areas of the Rat Heart. Cardiovasc. Drugs Ther. 26, 467–478. 10.1007/s10557-012-6422-0 23179953

[B17] Charlton A. Garzarella J. Jandeleit-Dahm K. a. M. Jha J. C. (2020). Oxidative Stress and Inflammation in Renal and Cardiovascular Complications of Diabetes. Biology 10, 18. 10.3390/biology10010018 33396868 PMC7830433

[B18] Chatuphonprasert W. Lao-Ong T. Jarukamjorn K. (2013). Improvement of Superoxide Dismutase and Catalase in Streptozotocin-Nicotinamide-Induced Type 2-Diabetes in Mice by Berberine and Glibenclamide. Pharm. Biol. 1, 1. 10.3109/13880209.2013.839714 24188560

[B19] Cheng F. Wang Y. Li J. Su C. Wu F. Xia W. H. (2013). Berberine Improves Endothelial Function by Reducing Endothelial Microparticles-Mediated Oxidative Stress in Humans. Int. J. Cardiol. 167, 936–942. 10.1016/j.ijcard.2012.03.090 22465347

[B20] Cheng H. Liu J. Tan Y. Feng W. Peng C. (2021). Interactions Between Gut Microbiota and Berberine, a Necessary Procedure to Understand the Mechanisms of Berberine. J. Pharm. Anal. 1, 1. 10.1016/j.jpha.2021.10.003 PMC9463479 36105164

[B21] Cheng W. L. Li S. J. Lee T. I. Lee T. W. Chung C. C. Kao Y. H. (2021). Sugar Fructose Triggers Gut Dysbiosis and Metabolic Inflammation with Cardiac Arrhythmogenesis. Biomedicines 9, 728. 10.3390/biomedicines9070728 34201938 PMC8301417

[B22] Chi J. F. Chu S. H. Lee C. S. Chou N. K. Su M. J. (1996). Mechanical and Electrophysiological Effects of 8-oxoberberine (JKL1073A) on Atrial Tissue. Br. J. Pharmacol. 118, 503–512. 10.1111/j.1476-5381.1996.tb15431.x 8762071 PMC1909730

[B23] Chi J. F. Chu S. H. Lee C. S. Su M. J. (1997). Effects of 8-Oxoberberine on Sodium Current in Rat Ventricular and Human Atrial Myocytes. Can. J. Cardiol. 13, 1103–1110. 9413244

[B24] Cicero A. F. Rovati L. C. Setnikar I. (2007). Eulipidemic Effects of Berberine Administered Alone or in Combination with Other Natural Cholesterol-Lowering Agents. A Single-Blind Clinical Investigation. Arzneimittelforschung 57, 26–30. 10.1055/s-0031-1296582 17341006

[B25] Cicero A. F. G. Fogacci F. Bove M. Giovannini M. Veronesi M. Borghi C. (2019). Short-Term Effects of Dry Extracts of Artichokeand Berberis in Hypercholesterolemic Patients without Cardiovascular Disease. Am. J. Cardiol. 123, 588–591. 10.1016/j.amjcard.2018.11.018 30528419

[B26] Cipolletta C. Ryan K. E. Hanna E. V. Trimble E. R. (2005). Activation of Peripheral Blood CD14+ Monocytes Occurs in Diabetes. Diabetes 54, 2779–2786. 10.2337/diabetes.54.9.2779 16123369

[B27] Corpas F. J. Gupta D. K. Palma J. M. (2015). “Production Sites of Reactive Oxygen Species (ROS) in Organelles from Plant Cells,” in Reactive Oxygen Species and Oxidative Damage in Plants under Stress, 1–22.

[B28] Cross C. E. Halliwell B. Borish E. T. Pryor W. A. Ames B. N. Saul R. L. (1987). Oxygen Radicals and Human Disease. Ann. Intern. Med. 107, 526–545. 10.7326/0003-4819-107-4-526 3307585

[B29] Cui X. Ye L. Li J. Jin L. Wang W. Li S. (2018). Metagenomic and Metabolomic Analyses Unveil Dysbiosis of Gut Microbiota in Chronic Heart Failure Patients. Sci. Rep. 8, 635. 10.1038/s41598-017-18756-2 29330424 PMC5766622

[B30] D’addato S. Scandiani L. Mombelli G. Focanti F. Pelacchi F. Salvatori E. (2017). Effect of a Food Supplement Containing Berberine, Monacolin K, Hydroxytyrosol and Coenzyme Q10 on Lipid Levels: A Randomized, Double-Blind, Placebo Controlled Study. Drug Des. Devel Ther. 11, 1585–1592. 10.2147/DDDT.S128623 PMC5447697 28579756

[B31] Dai D. Z. (2006). CPU86017: A Novel Class III Antiarrhythmic Agent with Multiple Actions at Ion Channels. Cardiovasc. Drug Rev. 24, 101–115. 10.1111/j.1527-3466.2006.00101.x 16961724

[B32] Davidson S. M. Yellon D. M. Murphy M. P. Duchen M. R. (2012). Slow Calcium Waves and Redox Changes Precede Mitochondrial Permeability Transition Pore Opening in the Intact Heart during Hypoxia and Reoxygenation. Cardiovasc. Res. 93, 445–453. 10.1093/cvr/cvr349 22198507

[B33] Diguet N. Trammell S. A. J. Tannous C. Deloux R. Piquereau J. Mougenot N. (2018). Nicotinamide Riboside Preserves Cardiac Function in a Mouse Model of Dilated Cardiomyopathy. Circulation 137, 2256–2273. 10.1161/CIRCULATIONAHA.116.026099 29217642 PMC6954688

[B34] Doerries C. Grote K. Hilfiker-Kleiner D. Luchtefeld M. Schaefer A. Holland S. M. (2007). Critical Role of the NAD(P)H Oxidase Subunit P47phox for Left Ventricular Remodeling/dysfunction and Survival after Myocardial Infarction. Circ. Res. 100, 894–903. 10.1161/01.RES.0000261657.76299.ff 17332431

[B35] Drummond G. R. Selemidis S. Griendling K. K. Sobey C. G. (2011). Combating Oxidative Stress in Vascular Disease: NADPH Oxidases as Therapeutic Targets. Nat. Rev. Drug Discov. 10, 453–471. 10.1038/nrd3403 21629295 PMC3361719

[B36] Erickson J. R. Joiner M. L. Guan X. Kutschke W. Yang J. Oddis C. V. (2008). A Dynamic Pathway for Calcium-Independent Activation of CaMKII by Methionine Oxidation. Cell 133, 462–474. 10.1016/j.cell.2008.02.048 18455987 PMC2435269

[B37] Fan X. Wang J. Hou J. Lin C. Bensoussan A. Chang D. (2015). Berberine Alleviates Ox-LDL Induced Inflammatory Factors by Up-Regulation of Autophagy via AMPK/mTOR Signaling Pathway. J. Transl Med. 13, 92. 10.1186/s12967-015-0450-z 25884210 PMC4365560

[B38] Fatahian A. Haftcheshmeh S. M. Azhdari S. Farshchi H. K. Nikfar B. Momtazi-Borojeni A. A. (2020). Promising Anti-Atherosclerotic Effect of Berberine: Evidence from *In Vitro*, *In Vivo*, and Clinical Studies. Rev. Physiol. Biochem. Pharmacol. 178, 83–110. 10.1007/112_2020_42 32789786

[B39] Feng M. Zou Z. Zhou X. Hu Y. Ma H. Xiao Y. (2017). Comparative Effect of Berberine and its Derivative 8-cetylberberine on Attenuating Atherosclerosis in ApoE-/- Mice. Int. Immunopharmacol. 43, 195–202. 10.1016/j.intimp.2016.12.001 28024280

[B40] Feng R. Shou J. W. Zhao Z. X. He C. Y. Ma C. Huang M. (2015). Transforming Berberine into its Intestine-Absorbable Form by the Gut Microbiota. Sci. Rep. 5, 12155. 10.1038/srep12155 26174047 PMC4502414

[B41] Feng W. Ao H. Peng C. Yan D. (2019). Gut Microbiota, a New Frontier to Understand Traditional Chinese Medicines. Pharmacol. Res. 142, 176–191. 10.1016/j.phrs.2019.02.024 30818043

[B42] Feng W. Liu J. Ao H. Yue S. Peng C. (2020). Targeting Gut Microbiota for Precision Medicine: Focusing on the Efficacy and Toxicity of Drugs. Theranostics 10, 11278–11301. 10.7150/thno.47289 33042283 PMC7532689

[B43] Feng X. Sureda A. Jafari S. Memariani Z. Tewari D. Annunziata G. (2019). Berberine in Cardiovascular and Metabolic Diseases: From Mechanisms to Therapeutics. Theranostics 9, 1923–1951. 10.7150/thno.30787 31037148 PMC6485276

[B44] Ference B. A. Ginsberg H. N. Graham I. Ray K. K. Packard C. J. Bruckert E. (2017). Low-density Lipoproteins Cause Atherosclerotic Cardiovascular Disease. 1. Evidence from Genetic, Epidemiologic, and Clinical Studies. A Consensus Statement from the European Atherosclerosis Society Consensus Panel. Eur. Heart J. 38, 2459–2472. 10.1093/eurheartj/ehx144 28444290 PMC5837225

[B45] Fouda M. A. Ghovanloo M. R. Ruben P. C. (2020). Cannabidiol Protects against High Glucose-Induced Oxidative Stress and Cytotoxicity in Cardiac Voltage-Gated Sodium Channels. Br. J. Pharmacol. 177, 2932–2946. 10.1111/bph.15020 32077098 PMC7279989

[B46] Fouda M. A. Ruben P. C. (2021). Protein Kinases Mediate Anti-inflammatory Effects of Cannabidiol and Estradiol against High Glucose in Cardiac Sodium Channels. Front. Pharmacol. 12, 668657. 10.3389/fphar.2021.668657 33995099 PMC8115126

[B47] Gan X. T. Ettinger G. Huang C. X. Burton J. P. Haist J. V. Rajapurohitam V. (2014). Probiotic Administration Attenuates Myocardial Hypertrophy and Heart Failure after Myocardial Infarction in the Rat. Circ. Heart Fail. 7, 491–499. 10.1161/CIRCHEARTFAILURE.113.000978 24625365

[B48] Gao J. Tang Y. Q. Dai D. Z. Cheng Y. S. Zhang G. L. Zhang C. (2012). Raisanberine Protected Pulmonary Arterial Rings and Cardiac Myocytes of Rats against Hypoxia Injury by Suppressing NADPH Oxidase and Calcium Influx. Acta Pharmacol. Sin 33, 625–634. 10.1038/aps.2012.7 22555370 PMC4010357

[B49] Girard S. A. Bah T. M. Kaloustian S. Lada-Moldovan L. Rondeau I. Tompkins T. A. (2009). Lactobacillus Helveticus and Bifidobacterium Longum Taken in Combination Reduce the Apoptosis Propensity in the Limbic System after Myocardial Infarction in a Rat Model. Br. J. Nutr. 102, 1420–1425. 10.1017/S0007114509990766 19563693

[B50] Halliwell B. Gutteridge J. M. Cross C. E. (1992). Free Radicals, Antioxidants, and Human Disease: where Are We Now? J. Lab. Clin. Med. 119, 598–620. 1593209

[B51] Hao P. Jiang F. Cheng J. Ma L. Zhang Y. Zhao Y. (2017). Traditional Chinese Medicine for Cardiovascular Disease: Evidence and Potential Mechanisms. J. Am. Coll. Cardiol. 69, 2952–2966. 10.1016/j.jacc.2017.04.041 28619197

[B52] Hershberger K. A. Martin A. S. Hirschey M. D. (2017). Role of NAD+ and Mitochondrial Sirtuins in Cardiac and Renal Diseases. Nat. Rev. Nephrol. 13, 213–225. 10.1038/nrneph.2017.5 28163307 PMC5508210

[B53] Howe C. J. Lahair M. M. Mccubrey J. A. Franklin R. A. (2004). Redox Regulation of the Calcium/calmodulin-dependent Protein Kinases. J. Biol. Chem. 279, 44573–44581. 10.1074/jbc.M404175200 15294913

[B54] Hsieh Y. S. Kuo W. H. Lin T. W. Chang H. R. Lin T. H. Chen P. N. (2007). Protective Effects of Berberine against Low-Density Lipoprotein (LDL) Oxidation and Oxidized LDL-Induced Cytotoxicity on Endothelial Cells. J. Agric. Food Chem. 55, 10437–10445. 10.1021/jf071868c 18001034

[B55] Hsu C. P. Oka S. Shao D. Hariharan N. Sadoshima J. (2009). Nicotinamide Phosphoribosyltransferase Regulates Cell Survival through NAD+ Synthesis in Cardiac Myocytes. Circ. Res. 105, 481–491. 10.1161/CIRCRESAHA.109.203703 19661458 PMC2765790

[B56] Huang W. M. Yan H. Jin J. M. Yu C. Zhang H. (1992). Beneficial Effects of Berberine on Hemodynamics during Acute Ischemic Left Ventricular Failure in Dogs. Chin. Med. J. 105, 1014–1019. 1299549

[B57] Huang Y. Mironova M. Lopes-Virella M. F. (1999). Oxidized LDL Stimulates Matrix Metalloproteinase-1 Expression in Human Vascular Endothelial Cells. Arterioscler Thromb. Vasc. Biol. 19, 2640–2647. 10.1161/01.atv.19.11.2640 10559006

[B58] Huang Z. Cai X. Li S. Zhou H. Chu M. Shan P. (2013). Berberine-Attenuated Monocyte Adhesion to Endothelial Cells Induced by Oxidized Low-Density Lipoprotein via Inhibition of Adhesion Molecule Expression. Mol. Med. Rep. 7, 461–465. 10.3892/mmr.2012.1236 23241897

[B59] Hulsmans M. Holvoet P. (2010). The Vicious Circle Between Oxidative Stress and Inflammation in Atherosclerosis. J. Cel Mol Med 14, 70–78. 10.1111/j.1582-4934.2009.00978.x PMC3837590 19968738

[B60] Jeong H. W. Hsu K. C. Lee J. W. Ham M. Huh J. Y. Shin H. J. (2009). Berberine Suppresses Proinflammatory Responses Through AMPK Activation in Macrophages. Am. J. Physiol. Endocrinol. Metab. 296, E955–E964. 10.1152/ajpendo.90599.2008 19208854

[B61] Jeroudi M. O. Hartley C. J. Bolli R. (1994). Myocardial Reperfusion Injury: Role of Oxygen Radicals and Potential Therapy with Antioxidants. Am. J. Cardiol. 73, 2B–7B. 10.1016/0002-9149(94)90257-7 8141076

[B62] Jiang Y. Huang K. Lin X. Chen Q. Lin S. Feng X. (2017). Berberine Attenuates NLRP3 Inflammasome Activation in Macrophages to Reduce the Secretion of Interleukin-1β. Ann. Clin. Lab. Sci. 47, 720–728. 29263046

[B63] Ju J. Li J. Lin Q. Xu H. (2018). Efficacy and Safety of Berberine for Dyslipidaemias: A Systematic Review and Meta-Analysis of Randomized Clinical Trials. Phytomedicine 50, 25–34. 10.1016/j.phymed.2018.09.212 30466986

[B64] Kamo T. Akazawa H. Suda W. Saga-Kamo A. Shimizu Y. Yagi H. (2017). Dysbiosis and Compositional Alterations with Aging in the Gut Microbiota of Patients with Heart Failure. PLoS One 12, e0174099. 10.1371/journal.pone.0174099 28328981 PMC5362204

[B65] Karam B. S. Chavez-Moreno A. Koh W. Akar J. G. Akar F. G. (2017). Oxidative Stress and Inflammation as central Mediators of Atrial Fibrillation in Obesity and Diabetes. Cardiovasc. Diabetol. 16, 120. 10.1186/s12933-017-0604-9 28962617 PMC5622555

[B66] Kattoor A. J. Pothineni N. V. K. Palagiri D. Mehta J. L. (2017). Oxidative Stress in Atherosclerosis. Curr. Atheroscler. Rep. 19, 42. 10.1007/s11883-017-0678-6 28921056

[B67] Kim Y. M. Ha Y. M. Jin Y. C. Shi L. Y. Lee Y. S. Kim H. J. (2009). Palmatine from Coptidis Rhizoma Reduces Ischemia-Reperfusion-Mediated Acute Myocardial Injury in the Rat. Food Chem. Toxicol. 47, 2097–2102. 10.1016/j.fct.2009.05.031 19497345

[B68] Kleemann R. Zadelaar S. Kooistra T. (2008). Cytokines and Atherosclerosis: A Comprehensive Review of Studies in Mice. Cardiovasc. Res. 79, 360–376. 10.1093/cvr/cvn120 18487233 PMC2492729

[B69] Ko W. H. Yao X. Q. Lau C. W. Law W. I. Chen Z. Y. Kwok W. (2000). Vasorelaxant and Antiproliferative Effects of Berberine. Eur. J. Pharmacol. 399, 187–196. 10.1016/s0014-2999(00)00339-3 10884519

[B70] Ko Y. J. Lee J. S. Park B. C. Shin H. M. Kim J. A. (2007). Inhibitory Effects of Zoagumhwan Water Extract and Berberine on Angiotensin II-Induced Monocyte Chemoattractant Protein (MCP)-1 Expression and Monocyte Adhesion to Endothelial Cells. Vascul Pharmacol. 47, 189–196. 10.1016/j.vph.2007.06.004 17631057

[B71] Korantzopoulos P. Letsas K. Fragakis N. Tse G. Liu T. (2018). Oxidative Stress and Atrial Fibrillation: an Update. Free Radic. Res. 52, 1199–1209. 10.1080/10715762.2018.1500696 30003814

[B72] Kummen M. Mayerhofer C. C. K. Vestad B. Broch K. Awoyemi A. Storm-Larsen C. (2018). Gut Microbiota Signature in Heart Failure Defined from Profiling of 2 Independent Cohorts. J. Am. Coll. Cardiol. 71, 1184–1186. 10.1016/j.jacc.2017.12.057 29519360

[B73] Kuznetsova T. Prange K. H. M. Glass C. K. De Winther M. P. J. (2020). Transcriptional and Epigenetic Regulation of Macrophages in Atherosclerosis. Nat. Rev. Cardiol. 17, 216–228. 10.1038/s41569-019-0265-3 31578516 PMC7770754

[B74] Lam V. Su J. Koprowski S. Hsu A. Tweddell J. S. Rafiee P. (2012). Intestinal Microbiota Determine Severity of Myocardial Infarction in Rats. FASEB J. 26, 1727–1735. 10.1096/fj.11-197921 22247331 PMC3316900

[B75] Lam V. Su J. Hsu A. Gross G. J. Salzman N. H. Baker J. E. (2016). Intestinal Microbial Metabolites are Linked to Severity of Myocardial Infarction in Rats. PLoS One 11, e0160840. 10.1371/journal.pone.0160840 27505423 PMC4978455

[B76] Lan J. Zhao Y. Dong F. Yan Z. Zheng W. Fan J. (2015). Meta-Analysis of the Effect and Safety of Berberine in the Treatment of Type 2 Diabetes Mellitus, Hyperlipemia and Hypertension. J. Ethnopharmacol 161, 69–81. 10.1016/j.jep.2014.09.049 25498346

[B77] Lassègue B. San Martín A. Griendling K. K. (2012). Biochemistry, Physiology, and Pathophysiology of NADPH Oxidases in the Cardiovascular System. Circ. Res. 110, 1364–1390. 10.1161/CIRCRESAHA.111.243972 22581922 PMC3365576

[B78] Lau C. W. Yao X. Q. Chen Z. Y. Ko W. H. Huang Y. (2001). Cardiovascular Actions of Berberine. Cardiovasc. Drug Rev. 19, 234–244. 10.1111/j.1527-3466.2001.tb00068.x 11607041

[B79] Li H. He C. Wang J. Li X. Yang Z. Sun X. (2016). Berberine Activates Peroxisome Proliferator-Activated Receptor Gamma to Increase Atherosclerotic Plaque Stability in Apoe-/- Mice with Hyperhomocysteinemia. J. Diabetes Investig. 7, 824–832. 10.1111/jdi.12516 PMC5089944 27181586

[B80] Li N. Yang L. Dai D. Z. Wang Q. J. Dai Y. (2008). Chiral Separation of Racemate CPU86017, an Anti-arrhythmic Agent, Produces Stereoisomers Possessing Favourable Ion Channel Blockade and Less Alpha-Adrenoceptor Antagonism. Clin. Exp. Pharmacol. Physiol. 35, 643–650. 10.1111/j.1440-1681.2007.04854.x 18177475

[B81] Li Y. Chen X. Liu H. Luo F. Li G. (2009). Effects of Ginseng Total Saponins with Berberine on Plasma Brain Natriuretic Peptide and Ca2+ Concentration in Experimental Rats with Chronic Congestive Heart Failure. Zhongguo Zhong Yao Za Zhi 34, 324–327. 19445159

[B82] Li Y. Wang P. Chai M. J. Yang F. Li H. S. Zhao J. (2016). Effects of Berberine on Serum Inflammatory Factors and Carotid Atherosclerotic Plaques in Patients with Acute Cerebral Ischemic Stroke. Zhongguo Zhong Yao Za Zhi 41, 4066–4071. 10.4268/cjcmm20162128 28929697

[B83] Li Y. H. Yang P. Kong W. J. Wang Y. X. Hu C. Q. Zuo Z. Y. (2009). Berberine Analogues as a Novel Class of the Low-Density-Lipoprotein Receptor Up-Regulators: Synthesis, Structure-Activity Relationships, and Cholesterol-Lowering Efficacy. J. Med. Chem. 52, 492–501. 10.1021/jm801157z 19090767

[B84] Li Z. Geng Y. N. Jiang J. D. Kong W. J. (2014). Antioxidant and Anti-Inflammatory Activities of Berberine in the Treatment of Diabetes Mellitus. Evid. Based Complement. Alternat. Med. 2014, 289264. 10.1155/2014/289264 24669227 PMC3942282

[B85] Liang X. Zhang Z. Lv Y. Tong L. Liu T. Yi H. (2020). Reduction of Intestinal Trimethylamine by Probiotics Ameliorated Lipid Metabolic Disorders Associated with Atherosclerosis. Nutrition 79-80, 110941. 10.1016/j.nut.2020.110941 32858376

[B86] Liu D. Q. Chen S. P. Sun J. Wang X. M. Chen N. Zhou Y. Q. (2019). Berberine Protects against Ischemia-Reperfusion Injury: A Review of Evidence from Animal Models and Clinical Studies. Pharmacol. Res. 148, 104385. 10.1016/j.phrs.2019.104385 31400402

[B87] Liu J. C. Chan P. Chen Y. J. Tomlinson B. Hong S. H. Cheng J. T. (1999). The Antihypertensive Effect of the Berberine Derivative 6-Protoberberine in Spontaneously Hypertensive Rats. Pharmacology 59, 283–289. 10.1159/000028331 10575322

[B88] Liu J. Tan Y. Cheng H. Zhang D. Feng W. Peng C. (2022). Functions of Gut Microbiota Metabolites, Current Status and Future Perspectives.Pdf. Aging Dis. 1, 1. 10.14336/AD.2022.0104 PMC9286904 35855347

[B89] Liu L. Liu J. Huang Z. Yu X. Zhang X. Dou D. (2015). Berberine Improves Endothelial Function by Inhibiting Endoplasmic Reticulum Stress in the Carotid Arteries of Spontaneously Hypertensive Rats. Biochem. Biophys. Res. Commun. 458, 796–801. 10.1016/j.bbrc.2015.02.028 25686503

[B90] Liu M. Gu L. Sulkin M. S. Liu H. Jeong E. M. Greener I. (2013). Mitochondrial Dysfunction Causing Cardiac Sodium Channel Downregulation in Cardiomyopathy. J. Mol. Cell Cardiol. 54, 25–34. 10.1016/j.yjmcc.2012.10.011 23123323 PMC3595554

[B91] Liu M. Liu H. Dudley S. C. Jr (2010). Reactive Oxygen Species Originating from Mitochondria Regulate the Cardiac Sodium Channel. Circ. Res. 107, 967–974. 10.1161/CIRCRESAHA.110.220673 20724705 PMC2955818

[B92] Liu M. Sanyal S. Gao G. Gurung I. S. Zhu X. Gaconnet G. (2009). Cardiac Na+ Current Regulation by Pyridine Nucleotides. Circ. Res. 105, 737–745. 10.1161/CIRCRESAHA.109.197277 19745168 PMC2773656

[B93] Liu Y. F. Wen C. Y. Chen Z. Wang Y. Huang Y. Tu S. H. (2016). Effects of Berberine on NLRP3 and IL-1β Expressions in Monocytic THP-1 Cells with Monosodium Urate Crystals-Induced Inflammation. Biomed. Res. Int. 2016, 2503703. 10.1155/2016/2503703 27689075 PMC5027325

[B94] Liu Z. Liu H. Y. Zhou H. Zhan Q. Lai W. Zeng Q. (2017). Moderate-Intensity Exercise Affects Gut Microbiome Composition and Influences Cardiac Function in Myocardial Infarction Mice. Front. Microbiol. 8, 1687. 10.3389/fmicb.2017.01687 28919891 PMC5585143

[B95] Ma J. Y. Feng R. Tan X. S. Ma C. Shou J. W. Fu J. (2013). Excretion of Berberine and its Metabolites in Oral Administration in Rats. J. Pharm. Sci. 102, 4181–4192. 10.1002/jps.23718 24006193

[B96] Marazzi G. Cacciotti L. Pelliccia F. Iaia L. Volterrani M. Caminiti G. (2011). Long-term Effects of Nutraceuticals (Berberine, Red Yeast Rice, Policosanol) in Elderly Hypercholesterolemic Patients. Adv. Ther. 28, 1105–1113. 10.1007/s12325-011-0082-5 22113535

[B97] Marin-Neto J. A. Maciel B. C. Secches A. L. Gallo Júnior L. (1988). Cardiovascular Effects of Berberine in Patients with Severe Congestive Heart Failure. Clin. Cardiol. 11, 253–260. 10.1002/clc.4960110411 3365876

[B98] Mccafferty K. Byrne C. Yaqoob M. (2012). Intestinal Microbiota Determine Severity of Myocardial Infarction in Rats. FASEB J. 26, 4388–4389. 10.1096/fj.12-1102LTR 23118150

[B99] Mericskay M. (2016). Nicotinamide Adenine Dinucleotide Homeostasis and Signalling in Heart Disease: Pathophysiological Implications and Therapeutic Potential. Arch. Cardiovasc. Dis. 109, 207–215. 10.1016/j.acvd.2015.10.004 26707577

[B100] Minhas K. M. Saraiva R. M. Schuleri K. H. Lehrke S. Zheng M. Saliaris A. P. (2006). Xanthine Oxidoreductase Inhibition Causes Reverse Remodeling in Rats with Dilated Cardiomyopathy. Circ. Res. 98, 271–279. 10.1161/01.RES.0000200181.59551.71 16357304

[B101] Minutoli L. Puzzolo D. Rinaldi M. Irrera N. Marini H. Arcoraci V. (2016). ROS-Mediated NLRP3 Inflammasome Activation in Brain, Heart, Kidney, and Testis Ischemia/Reperfusion Injury. Oxid Med. Cell Longev. 2016, 2183026. 10.1155/2016/2183026 27127546 PMC4835650

[B102] Mirhadi E. Rezaee M. Malaekeh-Nikouei B. (2018). Nano Strategies for Berberine Delivery, a Natural Alkaloid of Berberis. Biomed. Pharmacother. 104, 465–473. 10.1016/j.biopha.2018.05.067 29793179

[B103] Murphy M. P. (2009). How Mitochondria Produce Reactive Oxygen Species. Biochem. J. 417, 1–13. 10.1042/BJ20081386 19061483 PMC2605959

[B104] Nattel S. Dobrev D. (2016). Electrophysiological and Molecular Mechanisms of Paroxysmal Atrial Fibrillation. Nat. Rev. Cardiol. 13, 575–590. 10.1038/nrcardio.2016.118 27489190

[B105] Neag M. A. Mocan A. Echeverría J. Pop R. M. Bocsan C. I. Crişan G. (2018). Berberine: Botanical Occurrence, Traditional Uses, Extraction Methods, and Relevance in Cardiovascular, Metabolic, Hepatic, and Renal Disorders. Front. Pharmacol. 9, 557. 10.3389/fphar.2018.00557 30186157 PMC6111450

[B106] Palomeque J. Sapia L. Valverde C. Rueda O. V. Salas M. Mattiazzi A. (2008). CaMKII Mediates Angiotensin II-Induced Cardiomyocytes Apoptosis: Role of Ca2+, ROS and p38MAPK. J. Mol. Cell Cardiol. 44, 764–765. 10.1016/j.yjmcc.2008.02.128

[B107] Pang B. Yu X. T. Zhou Q. Zhao T. Y. Wang H. Gu C. J. (2015). Effect of Rhizoma Coptidis (Huang Lian) on Treating Diabetes Mellitus. Evid. Based Complement Alternat Med. 2015, 921416. 10.1155/2015/921416 26508987 PMC4609856

[B108] Pillai J. B. Isbatan A. Imai S. Gupta M. P. (2005). Poly(ADP-ribose) Polymerase-1-Dependent Cardiac Myocyte Cell Death during Heart Failure is Mediated by NAD+ Depletion and Reduced Sir2alpha Deacetylase Activity. J. Biol. Chem. 280, 43121–43130. 10.1074/jbc.M506162200 16207712

[B109] Pisciotta L. Bellocchio A. Bertolini S. (2012). Nutraceutical Pill Containing Berberine versus Ezetimibe on Plasma Lipid Pattern in Hypercholesterolemic Subjects and its Additive Effect in Patients with Familial Hypercholesterolemia on Stable Cholesterol-Lowering Treatment. Lipids Health Dis. 11, 123. 10.1186/1476-511X-11-123 22998978 PMC3477057

[B110] Pizzino G. Irrera N. Cucinotta M. Pallio G. Mannino F. Arcoraci V. (2017). Oxidative Stress: Harms and Benefits for Human Health. Oxid Med. Cell Longev. 2017, 8416763. 10.1155/2017/8416763 28819546 PMC5551541

[B111] Qi M. Y. Feng Y. Dai D. Z. Li N. Cheng Y. S. Dai Y. (2010). CPU86017, a Berberine Derivative, Attenuates Cardiac Failure Through Normalizing Calcium Leakage and Downregulated Phospholamban and Exerting Antioxidant Activity. Acta Pharmacol. Sin 31, 165–174. 10.1038/aps.2009.180 20139899 PMC4002834

[B112] Qin-Wei Z. Yong-Guang L. I. (2016). Berberine Attenuates Myocardial Ischemia Reperfusion Injury by Suppressing the Activation of PI3K/AKT Signaling. Exp. Ther. Med. 11, 978–984. 10.3892/etm.2016.3018 26998023 PMC4774358

[B113] Qing Y. Dong X. Hongli L. Yanhui L. (2018). Berberine Promoted Myocardial protection of Postoperative Patients through Regulating Myocardial Autophagy. Biomed. Pharmacother. 105, 1050–1053. 10.1016/j.biopha.2018.06.088 30021340

[B114] Rajamohan S. B. Pillai V. B. Gupta M. Sundaresan N. R. Birukov K. G. Samant S. (2009). SIRT1 Promotes Cell Survival under Stress by Deacetylation-dependent Deactivation of poly(ADP-Ribose) Polymerase 1. Mol. Cell Biol. 29, 4116–4129. 10.1128/MCB.00121-09 19470756 PMC2715814

[B115] Sack M. N. Fyhrquist F. Y. Saijonmaa O. J. Fuster V. Kovacic J. C. (2017). Basic Biology of Oxidative Stress and the Cardiovascular System: Part 1 of a 3-Part Series. J. Am. Coll. Cardiol. 70, 196–211. 10.1016/j.jacc.2017.05.034 28683968 PMC5551687

[B116] Sánchez-Chapula J. (1996). Increase in Action Potential Duration and Inhibition of the Delayed Rectifier Outward Current Ik by Berberine in Cat Ventricular Myocytes. Br. J. Pharmacol. 117, 1427–1434. 10.1111/j.1476-5381.1996.tb15302.x 8730735 PMC1909453

[B117] Sena C. M. Pereira A. M. Seiça R. (2013). Endothelial Dysfunction - A Major Mediator of Diabetic Vascular Disease. Biochim. Biophys. Acta 1832, 2216–2231. 10.1016/j.bbadis.2013.08.006 23994612

[B118] Senoner T. Dichtl W. (2019). Oxidative Stress in Cardiovascular Diseases: Still a Therapeutic Target? Nutrients 11, 2090. 10.3390/nu11092090 PMC6769522 31487802

[B119] Shaffer J. E. (1985). Inotropic and Chronotropic Activity of Berberine on Isolated guinea Pig Atria. J. Cardiovasc. Pharmacol. 7, 307–315. 10.1097/00005344-198503000-00016 2581085

[B120] Shang W. Liu J. Yu X. Zhao J. (2010). Effects of Berberine on Serum Levels of Inflammatory Factors and Inflammatory Signaling Pathway in Obese Mice Induced by High Fat Diet. Zhongguo Zhong Yao Za Zhi 35, 1474–1477. 20822024

[B121] Shi Y. Hu J. Geng J. Hu T. Wang B. Yan W. (2018). Berberine Treatment Reduces Atherosclerosis by Mediating Gut Microbiota in apoE-/- Mice. Biomed. Pharmacother. 107, 1556–1563. 10.1016/j.biopha.2018.08.148 30257374

[B122] Smith S. C. Jr. Collins A. Ferrari R. Holmes D. R. Jr. Logstrup S. Mcghie D. V. (2012). Our Time: A Call to Save Preventable Death from Cardiovascular Disease (Heart Disease and Stroke). J. Am. Coll. Cardiol. 60, 2343–2348. 10.1016/j.jacc.2012.08.962 22995536

[B123] Suadoni M. T. Atherton I. (2021). Berberine for the Treatment of Hypertension: A Systematic Review. Complement. Ther. Clin. Pract. 42, 101287. 10.1016/j.ctcp.2020.101287 33461163

[B124] Sun Y. Lu Y. Saredy J. Wang X. Drummer Iv C. Shao Y. (2020). ROS Systems Are a New Integrated Network for Sensing Homeostasis and Alarming Stresses in Organelle Metabolic Processes. Redox Biol. 37, 101696. 10.1016/j.redox.2020.101696 32950427 PMC7767745

[B125] Swaminathan P. D. Purohit A. Soni S. Voigt N. Singh M. V. Glukhov A. V. (2011). Oxidized CaMKII Causes Cardiac Sinus Node Dysfunction in Mice. J. Clin. Invest. 121, 3277–3288. 10.1172/JCI57833 21785215 PMC3223923

[B126] Takimoto E. Champion H. C. Li M. Ren S. Rodriguez E. R. Tavazzi B. (2005). Oxidant Stress from Nitric Oxide Synthase-3 Uncoupling Stimulates Cardiac Pathologic Remodeling from Chronic Pressure Load. J. Clin. Invest. 115, 1221–1231. 10.1172/JCI21968 15841206 PMC1077169

[B127] Takimoto E. Kass D. A. (2007). Role of Oxidative Stress in Cardiac Hypertrophy and Remodeling. Hypertension 49, 241–248. 10.1161/01.HYP.0000254415.31362.a7 17190878

[B128] Tan H. L. Chan K. G. Pusparajah P. Duangjai A. Saokaew S. Mehmood Khan T. (2016). Rhizoma Coptidis: A Potential Cardiovascular Protective Agent. Front. Pharmacol. 7, 362. 10.3389/fphar.2016.00362 27774066 PMC5054023

[B129] Tan Y. Tang Q. Hu B. R. Xiang J. Z. (2007). Antioxidant Properties of Berberine on Cultured Rabbit Corpus Cavernosum Smooth Muscle Cells Injured by Hydrogen Peroxide. Acta Pharmacol. Sin 28, 1914–1918. 10.1111/j.1745-7254.2007.00705.x 18031604

[B130] Tang W. H. W. Li D. Y. Hazen S. L. (2019). Dietary Metabolism, the Gut Microbiome, and Heart Failure. Nat. Rev. Cardiol. 16, 137–154. 10.1038/s41569-018-0108-7 30410105 PMC6377322

[B131] Tian H. Kang Y. M. Gao H. L. Shi X. L. Fu L. Y. Li Y. (2019). Chronic Infusion of Berberine into the Hypothalamic Paraventricular Nucleus Attenuates Hypertension and Sympathoexcitation via the ROS/Erk1/2/iNOS Pathway. Phytomedicine 52, 216–224. 10.1016/j.phymed.2018.09.206 30599901

[B132] Tsutsui H. Kinugawa S. Matsushima S. (2009). Mitochondrial Oxidative Stress and Dysfunction in Myocardial Remodelling. Cardiovasc. Res. 81, 449–456. 10.1093/cvr/cvn280 18854381

[B133] Ukai T. Cheng C. P. Tachibana H. Igawa A. Zhang Z. S. Cheng H. J. (2001). Allopurinol Enhances the Contractile Response to Dobutamine and Exercise in Dogs with Pacing-Induced Heart Failure. Circulation 103, 750–755. 10.1161/01.cir.103.5.750 11156889

[B134] Van Der Meer P. Gaggin H. K. Dec G. W. (2019). ACC/AHA Versus ESC Guidelines on Heart Failure: JACC Guideline Comparison. J. Am. Coll. Cardiol. 73, 2756–2768. 10.1016/j.jacc.2019.03.478 31146820

[B135] Van Der Pol A. Van Gilst W. H. Voors A. A. Van Der Meer P. (2019). Treating Oxidative Stress in Heart Failure: Past, Present and Future. Eur. J. Heart Fail. 21, 425–435. 10.1002/ejhf.1320 30338885 PMC6607515

[B136] Violi F. Pastori D. Pignatelli P. Loffredo L. (2014). Antioxidants for Prevention of Atrial Fibrillation: a Potentially Useful Future Therapeutic Approach? A Review of the Literature and Meta-Analysis. Europace 16, 1107–1116. 10.1093/europace/euu040 24706090

[B137] Wang K. Feng X. Chai L. Cao S. Qiu F. (2017). The Metabolism of Berberine and its Contribution to the Pharmacological Effects. Drug Metab. Rev. 49, 139–157. 10.1080/03602532.2017.1306544 28290706

[B138] Wang L. Ma H. Xue Y. Shi H. Ma T. Cui X. (2018). Berberine Inhibits the Ischemia-Reperfusion Injury Induced Inflammatory Response and Apoptosis of Myocardial Cells through the Phosphoinositide 3-Kinase/RAC-α Serine/Threonine-Protein Kinase and Nuclear Factor-Κb Signaling Pathways. Exp. Ther. Med. 15, 1225–1232. 10.3892/etm.2017.5575 29403554 PMC5780743

[B139] Wang L. Peng L. Y. Wei G. H. Ge H. (2016). Therapeutic Effects of Berberine Capsule on Patients with Mild Hyperlipidemia. Zhongguo Zhong Xi Yi Jie He Za Zhi 36, 681–684. 27491226

[B140] Wang L. H. Li X. L. Li Q. Fu Y. Yu H. J. Sun Y. Q. (2012). Berberine Alleviates Ischemic Arrhythmias via Recovering Depressed I(to) and I(Ca) Currents in Diabetic Rats. Phytomedicine 19, 206–210. 10.1016/j.phymed.2011.11.002 22188769

[B141] Wang Y. Liu J. Ma A. Chen Y. (2015). Cardioprotective Effect of Berberine Against Myocardial Ischemia/reperfusion Injury via Attenuating Mitochondrial Dysfunction and Apoptosis. Int. J. Clin. Exp. Med. 8, 14513–14519. 26550442 PMC4613127

[B142] Wang Y. Tong Q. Shou J. W. Zhao Z. X. Li X. Y. Zhang X. F. (2017). Gut Microbiota-Mediated Personalized Treatment of Hyperlipidemia Using Berberine. Theranostics 7, 2443–2451. 10.7150/thno.18290 28744326 PMC5525748

[B143] Wang Y. X. Zheng Y. M. Zhou X. B. (1996). Inhibitory Effects of Berberine on ATP-Sensitive K+ Channels in Cardiac Myocytes. Eur. J. Pharmacol. 316, 307–315. 10.1016/s0014-2999(96)00663-2 8982702

[B144] Wang Y. Zhou X. Zhao D. Wang X. Gurley E. C. Liu R. (2020). Berberine Inhibits Free Fatty Acid and LPS-Induced Inflammation via Modulating ER Stress Response in Macrophages and Hepatocytes. PLoS One 15, e0232630. 10.1371/journal.pone.0232630 32357187 PMC7194368

[B145] Weissman D. Maack C. (2021). Redox Signaling in Heart Failure and Therapeutic Implications. Free Radic. Biol. Med. 171, 345–364. 10.1016/j.freeradbiomed.2021.05.013 34019933

[B146] Wu M. Yang S. Wang S. Cao Y. Zhao R. Li X. (2020). Effect of Berberine on Atherosclerosis and Gut Microbiota Modulation and Their Correlation in High-Fat Diet-Fed ApoE-/- Mice. Front. Pharmacol. 11, 223. 10.3389/fphar.2020.00223 32231564 PMC7083141

[B147] Xi H. Akishita M. Nagai K. Yu W. Hasegawa H. Eto M. (2007). Potent Free Radical Scavenger, Edaravone, Suppresses Oxidative Stress-Induced Endothelial Damage and Early Atherosclerosis. Atherosclerosis 191, 281–289. 10.1016/j.atherosclerosis.2006.05.040 16806227

[B148] Xu H. Y. Liu C. S. Huang C. L. Chen L. Zheng Y. R. Huang S. H. (2019). Nanoemulsion Improves Hypoglycemic Efficacy of Berberine by Overcoming its Gastrointestinal challenge. Colloids Surf. B Biointerf. 181, 927–934. 10.1016/j.colsurfb.2019.06.006 31382342

[B149] Xu J. H. Liu X. Z. Pan W. Zou D. J. (2017). Berberine Protects Against Diet-Induced Obesity through Regulating Metabolic Endotoxemia and Gut Hormone Levels. Mol. Med. Rep. 15, 2765–2787. 10.3892/mmr.2017.6321 28447763 PMC5428400

[B150] Xu T. Ding W. Ji X. Ao X. Liu Y. Yu W. (2019). Oxidative Stress in Cell Death and Cardiovascular Diseases. Oxid Med. Cell Longev. 2019, 9030563. 10.1155/2019/9030563 31781356 PMC6875219

[B151] Yang T. Wang R. Liu H. Wang L. Li J. Wu S. (2021). Berberine Regulates Macrophage Polarization Through IL-4-STAT6 Signaling Pathway in Helicobacter Pylori-Induced Chronic Atrophic Gastritis. Life Sci. 266, 118903. 10.1016/j.lfs.2020.118903 33340526

[B152] Yang X. An N. Zhong C. Guan M. Jiang Y. Li X. (2020). Enhanced Cardiomyocyte Reactive Oxygen Species Signaling Promotes Ibrutinib-Induced Atrial Fibrillation. Redox Biol. 30, 101432. 10.1016/j.redox.2020.101432 31986467 PMC6994714

[B153] Yang X.-J. Liu F. Feng N. Ding X. S. Chen Y. Zhu S. X. (2020). Berberine Attenuates Cholesterol Accumulation in Macrophage Foam Cells by Suppressing AP-1 Activity and Activation of the Nrf2/HO-1 Pathway. J. Cardiovasc. Pharmacol. 75, 45–53. 10.1097/FJC.0000000000000769 31895879

[B154] Ye F. Zhou Q. Tian L. Lei F. Feng D. (2017). The Protective Effect of Berberine Hydrochloride on LPS-Induced Osteoclastogenesis through Inhibiting TRAF6-Ca2+-Calcineurin-NFATcl Signaling Pathway. Mol. Med. Rep. 16, 6228–6233. 10.3892/mmr.2017.7338 28849049

[B155] Yousefian M. Shakour N. Hosseinzadeh H. Hayes A. W. Hadizadeh F. Karimi G. (2019). The Natural Phenolic Compounds as Modulators of NADPH Oxidases in Hypertension. Phytomedicine 55, 200–213. 10.1016/j.phymed.2018.08.002 30668430

[B156] Yu L. Li Q. Yu B. Yang Y. Jin Z. Duan W. (2016). Berberine Attenuates Myocardial Ischemia/Reperfusion Injury by Reducing Oxidative Stress and Inflammation Response: Role of Silent Information Regulator 1. Oxid Med. Cel Longev 2016, 1689602. 10.1155/2016/1689602 PMC4691633 26788242

[B157] Yuan X. Wang L. Bhat O. M. Lohner H. Li P. L. (2018). Differential Effects of Short Chain Fatty Acids on Endothelial Nlrp3 Inflammasome Activation and Neointima Formation: Antioxidant Action of Butyrate. Redox Biol. 16, 21–31. 10.1016/j.redox.2018.02.007 29475132 PMC5842312

[B158] Yue S. J. Liu J. Wang A. T. Meng X. T. Yang Z. R. Peng C. (2019). Berberine Alleviates Insulin Resistance by Reducing Peripheral Branched-Chain Amino Acids. Am. J. Physiol. Endocrinol. Metab. 316, E73–E85. 10.1152/ajpendo.00256.2018 30422704

[B159] Zeng X. H. Zeng X. J. Li Y. Y. (2003). Efficacy and Safety of Berberine for Congestive Heart Failure Secondary to Ischemic or Idiopathic Dilated Cardiomyopathy. Am. J. Cardiol. 92, 173–176. 10.1016/s0002-9149(03)00533-2 12860219

[B160] Zhan K. Y. Yu P. L. Liu C. H. Luo J. H. Yang W. (2016). Detrimental or Beneficial: the Role of TRPM2 in Ischemia/reperfusion Injury. Acta Pharmacol. Sin 37, 4–12. 10.1038/aps.2015.141 26725732 PMC4722978

[B161] Zhang B. Zhai M. Li B. Liu Z. Li K. Jiang L. (2018). Honokiol Ameliorates Myocardial Ischemia/Reperfusion Injury in Type 1 Diabetic Rats by Reducing Oxidative Stress and Apoptosis through Activating the SIRT1-Nrf2 Signaling Pathway. Oxid Med. Cell Longev. 2018, 3159801. 10.1155/2018/3159801 29675132 PMC5838504

[B162] Zhang L. S. Zhang J. H. Feng R. Jin X. Y. Yang F. W. Ji Z. C. (2019). Efficacy and Safety of Berberine Alone or Combined with Statins for the Treatment of Hyperlipidemia: A Systematic Review and Meta-Analysis of Randomized Controlled Clinical Trials. Am. J. Chin. Med. 47, 751–767. 10.1142/S0192415X19500393 31094214

[B163] Zhang M. Wang C. M. Li J. Meng Z. J. Wei S. N. Li J. (2013). Berberine Protects against Palmitate-Induced Endothelial Dysfunction: Involvements of Upregulation of AMPK and eNOS and Downregulation of NOX4. Mediators Inflamm. 2013, 260464. 10.1155/2013/260464 24385682 PMC3872165

[B164] Zhang T. T. Cui B. Dai D. Z. Su W. (2005). CPU 86017, P-Chlorobenzyltetrahydroberberine Chloride, Attenuates Monocrotaline-Induced Pulmonary Hypertension by Suppressing Endothelin Pathway. Acta Pharmacol. Sin 26, 1309–1316. 10.1111/j.1745-7254.2005.00214.x 16225752

[B165] Zhang X. D. Ren H. M. Liu L. (2008). Effects of Different Dose Berberine on Hemodynamic Parameters and [Ca2+]i of Cardiac Myocytes of Diastolic Heart Failure Rat Model. Zhongguo Zhong Yao Za Zhi 33, 818–821. 18589791

[B166] Zhang Y. Zhang S. Li B. Luo Y. Gong Y. Jin X. (2021). Gut Microbiota Dysbiosis Promotes Age-Related Atrial Fibrillation by Lipopolysaccharide and Glucose-Induced Activation of NLRP3-Inflammasome. Cardiovasc. Res. 1, cvab114. 10.1093/cvr/cvab114 33757127

[B167] Zhao D. Liu J. Wang M. Zhang X. Zhou M. (2019). Epidemiology of Cardiovascular Disease in China: Current Features and Implications. Nat. Rev. Cardiol. 16, 203–212. 10.1038/s41569-018-0119-4 30467329

[B168] Zhao G. L. Yu L. M. Gao W. L. Duan W. X. Jiang B. Liu X. D. (2016). Berberine Protects Rat Heart from Ischemia/reperfusion Injury via Activating JAK2/STAT3 Signaling and Attenuating Endoplasmic Reticulum Stress. Acta Pharmacol. Sin 37, 354–367. 10.1038/aps.2015.136 26806299 PMC4775848

[B169] Zhou H. Feng L. Xu F. Sun Y. Ma Y. Zhang X. (2017). Berberine Inhibits Palmitate-Induced NLRP3 Inflammasome Activation by Triggering Autophagy in Macrophages: A New Mechanism Linking Berberine to Insulin Resistance Improvement. Biomed. Pharmacother. 89, 864–874. 10.1016/j.biopha.2017.03.003 28282788

[B170] Zhou M. Wang H. Zhu J. Chen W. Wang L. Liu S. (2016). Cause-Specific Mortality for 240 Causes in China during 1990-2013: a Systematic Subnational Analysis for the Global Burden of Disease Study 2013. Lancet 387, 251–272. 10.1016/S0140-6736(15)00551-6 26510778

[B171] Zhou Z. W. Zheng H. C. Zhao L. F. Li W. Hou J. W. Yu Y. (2015). Effect of Berberine on Acetylcholine-Induced Atrial Fibrillation in Rabbit. Am. J. Transl Res. 7, 1450–1457. 26396675 PMC4568800

[B172] Zhu J. X. Tang D. Feng L. Zheng Z. G. Wang R. S. Wu A. G. (2013). Development of Self-Microemulsifying Drug Delivery System for Oral Bioavailability Enhancement of Berberine Hydrochloride. Drug Dev. Ind. Pharm. 39, 499–506. 10.3109/03639045.2012.683875 22563917

[B173] Zhu L. Zhang D. Zhu H. Zhu J. Weng S. Dong L. (2018). Berberine Treatment Increases Akkermansia in the Gut and Improves High-Fat Diet-Induced Atherosclerosis in Apoe-/- Mice. Atherosclerosis 268, 117–126. 10.1016/j.atherosclerosis.2017.11.023 29202334

[B174] Ziolo M. T. Mohler P. J. (2015). Defining the Role of Oxidative Stress in Atrial Fibrillation and Diabetes. J. Cardiovasc. Electrophysiol. 26, 223–225. 10.1111/jce.12560 25298131 PMC4323889

[B175] Zununi Vahed S. Barzegari A. Zuluaga M. Letourneur D. Pavon-Djavid G. (2018). Myocardial Infarction and Gut Microbiota: An Incidental Connection. Pharmacol. Res. 129, 308–317. 10.1016/j.phrs.2017.11.008 29133215

[B176] Zuo K. Li J. Li K. Hu C. Gao Y. Chen M. (2019). Disordered Gut Microbiota and Alterations in Metabolic Patterns Are Associated with Atrial Fibrillation. Gigascience 8, giz058. 10.1093/gigascience/giz058 31149718 PMC6543127

